# Validation of human sensory neurons derived from inducible pluripotent stem cells as a model for latent infection and reactivation by herpes simplex virus 1

**DOI:** 10.1128/mbio.01871-25

**Published:** 2025-08-18

**Authors:** Hyung Suk Oh, Shu-Fan Chou, Priya Raja, Jaehoon Shim, Biswajit Das, Jean M. Pesola, Nicolás Romero, Jennifer S. Lee, Alex Ng, Elizabeth D. Buttermore, George Church, Clifford J. Woolf, Donald M. Coen, David M. Knipe

**Affiliations:** 1 Department of Microbiology, Blavatnik Institute, Harvard Medical School 630058 , Boston, Massachusetts, USA; 2 F.M. Kirby Neurobiology Department, Department of Neurology, Boston Children's Hospital 548390, Boston, Massachusetts, USA; 3 Department of Biological Chemistry and Molecular Pharmacology, Blavatnik Institute, Harvard Medical School 630058 , Boston, Massachusetts, USA; 4 Department of Genetics, Blavatnik Institute, Harvard Medical School 630058 , Boston, Massachusetts, USA; 5 Wyss Institute for Biologically Inspired Engineering at Harvard University 465574, Boston, Massachusetts, USA; 6 Human Neuron Core, Rosamund Stone Zander Translational Neuroscience Center, Boston Children’s Hospital 24172 , Boston, Massachusetts, USA; Princeton University, Princeton, New Jersey, USA

**Keywords:** herpes simplex virus 1, latent infection, human sensory neurons

## Abstract

Herpes simplex virus 1 (HSV-1) undergoes primary acute lytic replication in mucosal epithelial cells and then spreads to establish latent infection in peripheral neurons including sensory and autonomic neurons. Latent HSV-1 reactivates to cause recurrent cold sores but can also cause more severe disease including keratitis and encephalitis. Animal models have been used extensively to study HSV-1 latent infection, while human sensory neuronal systems have been difficult to establish or make scalable. To address neuron-intrinsic mechanisms of HSV-1 latent infection, we developed a protocol to rapidly differentiate human-inducible pluripotent stem cells (hiPSCs) into sensory neurons. The differentiated neurons are excitable and express functional ion channels. We established conditions for latent infection with HSV-1 in these cells that show i) no infectious virus, ii) reduced lytic gene expression, iii) efficient latency-associated transcript expression, and iv) viral heterochromatin. Latent HSV-1 can be reactivated by previously known stimuli including forskolin and PI3Ki. Therefore, this scalable human iPSC-derived sensory neuron system is a promising model to explore mechanisms of HSV-1 latent infection in human neurons.

**IMPORTANCE:**

Herpes simplex viruses are prevalent human pathogens that cause significant morbidity and mortality in neonates and adults. They undergo acute infection in the mucosae and spread to peripheral neurons including sensory and autonomic neurons, where they establish lifelong latent infection. Reactivation from latency leads to recurrent infection and disease, including central nervous system infection that can be life-threatening. Experimental studies of HSV latency have mostly been conducted in animals, which may differ from the human situation. In this study, we establish a system for differentiation of human-inducible pluripotent stem cells into sensory neurons and for latent infection and reactivation by herpes simplex virus 1. This system will enable studies of the mechanism of HSV latent infection in human sensory neurons and therapeutic approaches to curtail it.

## INTRODUCTION

Herpes simplex virus (HSV) 1 and 2 are prevalent human pathogens causing significant morbidity and mortality in neonates and adults. After primary infection, HSV undergoes lytic replication in epithelial tissues, followed by spread to peripheral neurons including sensory and autonomic neurons where the virus establishes lifelong latent infection in humans. Lytic infection can cause herpetic cold sores and genital herpes. Primary infection by HSV or reactivation of latent HSV can lead to these and more severe diseases including herpes keratitis, meningitis, and encephalitis. In addition, HSV-2 infection can increase the risk of HIV acquisition (1–3). Although no approved vaccine for HSV-1 or −2 is currently available, several effective anti-HSV drugs can be used to treat lytic infection by targeting viral enzymes expressed during the lytic replication cycle. However, there is no treatment available for latent HSV infection. Therefore, further knowledge of the mechanisms of latent infection in human sensory neurons is needed to devise strategies to cure or treat latent infection or prevent reactivation.

Based on studies in animal models, following infection of the epithelium by HSV-1, it infects the axonal termini of sensory or autonomic neurons of ganglia. The nucleocapsid travels along the axon to the cell body that resides in sensory or autonomic ganglia such as trigeminal ganglia (TG), dorsal root ganglia (DRG), and superior cervical ganglion (SCG). There, the viral genome enters the neuronal nucleus, circularizes, and is silenced by cellular epigenetic mechanisms (4). The virus establishes a latent infection with abundant viral gene expression limited to products of the latency-associated transcript (LAT) locus (5–7), while lytic transcripts are expressed at low levels (8–18).

The latent HSV-1 genome is loaded with histones bearing facultative heterochromatin markers (19–21). Studies using chromatin immunoprecipitation (ChIP) analyses have demonstrated that, during lytic infection, input HSV-1 genomes are rapidly subjected to the assembly of nucleosomes and association with repressive heterochromatin markers histone 3 (H3) lysine 9-trimethylation (H3K9me3) and lysine 27-trimethylation (H3K27me3) within 1 to 2 hours post-infection (hpi), but the heterochromatin markers (H3K9me3 and H3K27me3 (or H3K27me2)) were rapidly decreased from 2 to 3 hpi (22, 23). During the establishment of latent infection *in vivo*, lytic gene promoters of the HSV-1 genome were shown to be associated with H3 and heterochromatin markers, and their associations increased over days 7–14 post-infection (19, 24). In addition, LAP, the promoter for LAT transcription, was also shown to be associated with H3 and H3K27me3, but there was no significant change of H3 or H3K27me3 association with LAP during the establishment of HSV-1 latency with or without acute replication *in vivo* (24).

Based on studies in rodent neuron culture models, reactivation involves alterations in certain neuronal signaling pathways (25) and the reversal of heterochromatin silencing by a phospho/methyl mechanism activated by a kinase pathway (26) induced by c-Jun N-terminal kinase kinase (JNK) pathways. Various stimuli have been shown to induce the reactivation of HSV-1 and HSV-2 from latent infection in various *in vivo* animal models and rodent neuron culture model systems. The efficiency of induced reactivation depended on stimuli, the model system and its genetic background, strains of virus, and cell types. Different stimuli target different pathways to induce reactivation of HSV (27, 28). Blocking TrkA signaling by depletion of nerve growth factor (NGF) or treatment with phosphoinositide 3 kinase (PI3K) inhibitors induced reactivation in an *in vitro* murine neuron culture system (25, 29). Stimulating transient receptor potential vanilloid 1 (TRPV1) by capsaicin or heat shock could reactivate latent HSV-1 (30). Hyperthermia induces reactivation *in vivo* (31). Various treatments including corticosteroids such as dexamethasone (32, 33), DNA damage (34), treatment with small molecules for epigenetic regulation (trichostatin A (TSA) and SP600125) (26), induction of cyclic AMP (35), BET domain inhibitors (36), or stimulating innate immune pathways (37) all induce reactivation of latent HSV-1 in murine neuronal cell culture systems. One study showed that IL-1β and reagents inducing action potentials trigger the reactivation in a murine neuron culture system (38).

Although these results have been highly valuable, their applicability to what occurs in humans and human sensory neurons is unclear. Important insights have come from primary human sensory neurons from autopsy specimens (39–42). However, these are extremely limited in supply and difficult at best to manipulate experimentally. Alternatively, there are human neuronal cell lines, but these are either derived from tumors and/or are not sensory neurons. Recently, latent infection and chromatin modifications in human neurons have been examined in v-*myc*-immortalized neuronal precursor Lund human mesencephalic (LUHMES) cells or neurons differentiated from them (43, 44), but differentiated LUHMES neurons are dopaminergic neurons originating from midbrain mesencephalon, which can show a different response to HSV-1 infection compared with sensory neurons (45).

A scalable human sensory neuron culture system that avoids the use of embryonic stem cells would be very useful to investigate more effectively lytic infection, establishment of latent infection, and reactivation in human sensory neurons. HSV-1 latent infection of sensory neurons has been defined as the lack of infectious virus in the cells but the ability to reactivate. Additional characteristics of latent infection are i) presence of viral DNA wrapped in nucleosomes with facultative heterochromatin markers, ii) expression of latency-associated transcripts (LATs), and iii) low levels of lytic gene expression. Here, we describe a system for the differentiation of human-inducible pluripotent stem cells (hiPSC)-derived sensory neurons, the validation of these cells as sensory neurons, and the use of these cells for latent infection by HSV-1 using these criteria.

## RESULTS

### Development and validation of iPSC-derived sensory neurons

**Fig 1:**
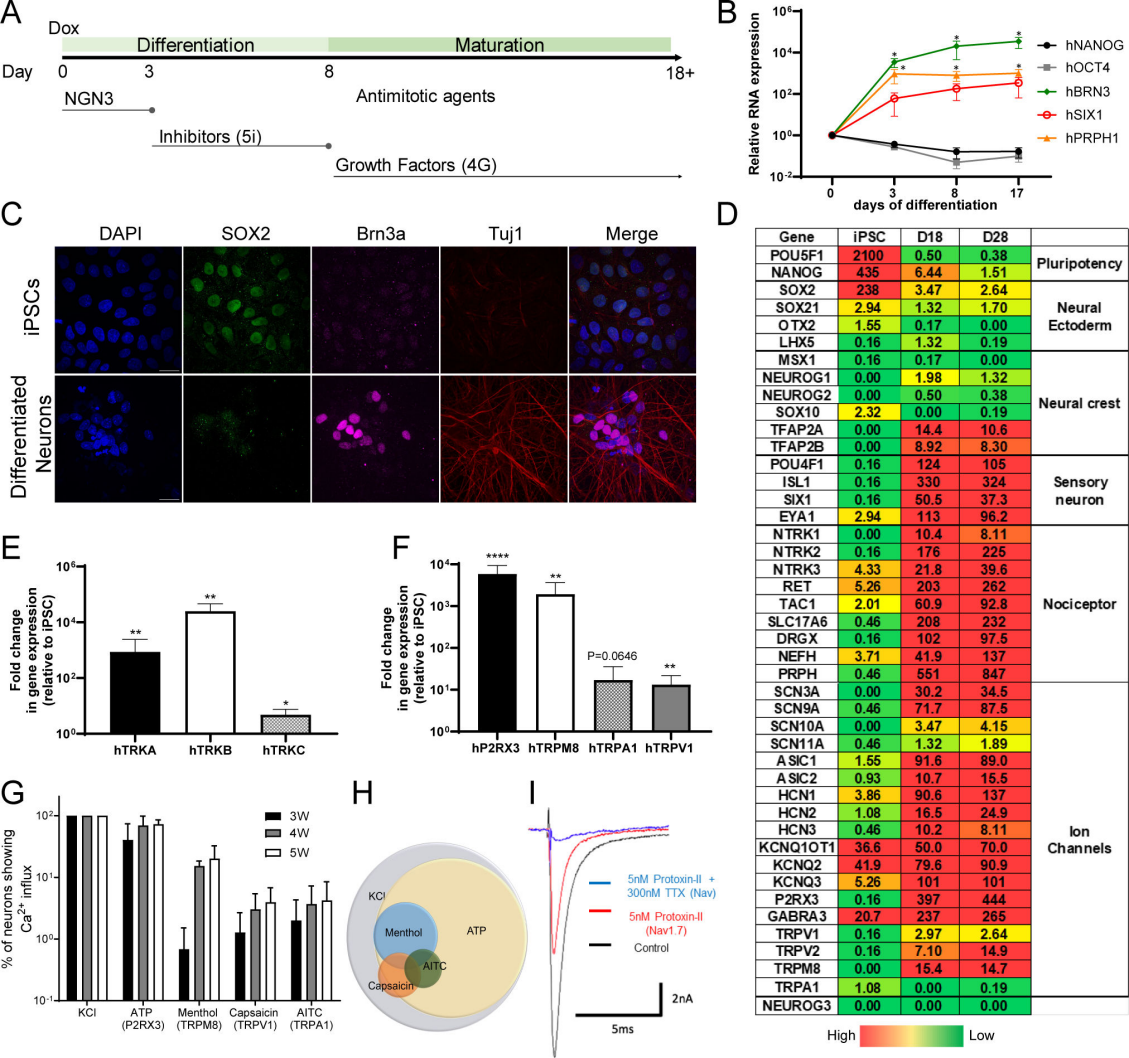
Differentiation and characterization of human iNGN3-derived neurons. (**A**) Schematic diagram of the protocol and timeline for iNGN3 differentiation and infection. (**B–F**) Gene expression profile of iPSC-derived neurons collected at different time points post-differentiation. (**B**) The values on the Y-axis represent the relative levels of transcripts encoding stem cells, neural progenitors, and sensory neuronal cell markers of differentiated cells versus iPSCs, as measured by RT-qPCR. (**C**) Expression of Sox2 (green), Brn3a (magenta), and Tuj1 (red) detected using immunofluorescence in 21-d differentiated neurons. DAPI (blue) was used to identify cell nuclei. Scale bar = 20 µm (**D**) Heat map of RNA-seq data from day 18 (D18) and day 28 (D28) post-differentiated neurons. The table uses a color gradient to indicate magnitude, the highest values (>10), next highest values (>2, <10), and the lowest values (<2). (**E and F**) Gene expressions of the human neurotrophin receptor TRK family (**E**) and nociceptor neuron-enriched cation channel receptors (**F**) were measured and compared with un-differentiated iPSCs at 17 days post-differentiation. (**G**) Measurement of agonist-stimulating calcium influx in 21- to 35-day differentiated neurons. (**H**) Venn diagram of subgroups of differentiated neurons responding to KCl, menthol, capsaicin, and AITC. (**I**) Action potential recording of TTX and protoxin-II response using D21 differentiated neurons. The graph shows the mean values and standard deviations of biological replicates. Statistical significance was evaluated by ratio paired *t*-tests, and multiplicity-adjusted *P* values were determined via the Holm-Šídák method. A family-wise alpha level of 0.05 was applied to each gene’s time course in which 3, 8, and 17 days were each compared to 0 day; only significant differences between the days are labeled with asterisks (**B**). A family-wise alpha level of 0.05 was applied to each figure (**E and F**). *N* ≥ 3, *: *P* < 0.05, **: *P* < 0.001, and ****: *P* < 0.0001.

To develop an *in vitro* human neuronal cell culture model for latent infection with HSV-1, we used human neurogenin 3 (NEUROG3) iPSC (iNGN3) derived from Personal Genome Project Participant 1 iPSCs (46). These cells harbor a doxycycline (Dox)-inducible NEUROG3 gene that drives differentiation into functional bipolar diverse subtypes of neurons within 4 days (d). To further specify the differentiation of NEUROG3 neurons into sensory neurons, we combined NGN3 induction with protocols using small molecules that drive sensory neuronal differentiation (45, 47–49). We induced NGN3 expression using Dox for 3 d (Fig. 1A) and then switched to five reprogramming small-molecule inhibitors (5i) for 5 days with a modification of a previously reported protocol (47–49). Then, we further promoted neuronal differentiation for ten or more days (maturation periods) in neuronal media supplemented with B27 or B27 plus, ascorbic acid, and four growth factors (4G medium containing brain-derived neurotrophic factor (BDNF), glial cell line-derived neurotrophic factor (GDNF), β-NGF, and neurotrophin-3 (NT-3)). During the maturation periods, we treated the cells with either mitomycin C (MMC) or 5-fluorodeoxyuridine (FUDR) (Fig. 1A) to eliminate any undifferentiated mitotic cells.

To validate the sensory neuronal differentiation, we measured specific transcript levels of stem cell, neural progenitor, and sensory neuronal cell markers at different time points post-differentiation using reverse transcription-quantitative polymerase chain reaction (RT-qPCR). Following NGN3 induction, we observed decreased levels of transcripts encoding the stem cell markers, NANOG and OCT4 (Fig. 1B), but dramatic increases in transcripts of peripheral sensory neuronal markers such as BRN3, PRPH1 (neurofilament), and SIX1, which reached a plateau from 8 to 17 days post-differentiation (Fig. 1B). We also stained the iNGN3 and differentiated cells with various antibodies specific for sensory neuronal markers Tuj1, Brn3a (Fig. 1C), as well as stem cell markers Sox2. Undifferentiated iNGN3 cells uniformly expressed Sox2, whereas the differentiated cells exhibited little or no expression of Sox2 (Fig. 1C) while Tuj1 and Brn3a (Fig. 1C) or Brn3a, Isl1, NeuN, TrkA, TrkB, TrkC, Nav1.7, Nav1.8, TRPM8, TRPV1, TRPA1, and peripherin were expressed at 21 days post-differentiation (Fig. S1A through F).

To further define the transcriptional profile during differentiation, we collected cellular RNA at 18 and 28 days post-differentiation and performed RNA sequencing (RNA-seq) analysis. While transcripts encoding stem cell and neural ectoderm markers were reduced by differentiation, the differentiated neurons showed increased levels of transcripts for transcription factors associated with development of sensory/periphery neurons (Fig. 1D). Multiple nociceptive neuronal markers and ion channels known to be expressed in sensory neurons were also substantially upregulated upon differentiation (Fig. 1D). We also performed RT-qPCR to confirm the expression of individual transcripts of neurotrophin receptors, and we found that all three neurotrophin receptors, TRKA, B, and C, were significantly upregulated in the differentiated neurons (Fig. 1E). Similarly, major ionotropic receptors and nociceptor ion channels, including P2R × 3, TRPM8, and TRPV1, were expressed at significantly higher levels, while TRPA1 (*P* = 0.0646) showed a moderate increase in these neurons at 21 days post-differentiation (Fig. 1F). From the gene expression profile, we concluded that this protocol efficiently and robustly reprograms iPSCs into diverse subtypes of primary sensory neurons including nociceptors, mechanoceptors, proprioceptors, thermoreceptors, and pruriceptors.

To validate the functions of ionotropic receptors in these cells, we imaged calcium responses with different pain-related noxious stimuli in 3-, 4-, and 5 week-differentiated neurons (Fig. 1G). All of the differentiated cells responded to KCl (Fig. 1G), and subpopulations of the cells responded to AITC (allyl isothiocyanate and TRPA1 agonist), capsaicin (TRPV1 agonist), or menthol (TRPM8 agonist) at 21 days post-differentiation, and the proportion of cells responding to these stimuli increased by 35 days post-differentiation (Fig. 1G and H). Interestingly, the fraction of cells responding to ATP (P2R × 3 expressing neurons) was higher than that to the other stimuli at 21 days post-differentiation. The results of the functional analysis with different stimuli (Fig. 1H) correlated well with the gene expression of corresponding receptors (Fig. 1D and F) in these neurons, demonstrating that the cells were a heterogeneous population of sensory neurons.

We also performed whole-cell patch-clamp (voltage-clamp) recordings to examine the function of the sodium channels detected in our RNA-seq data. A subpopulation of the differentiated sensory neurons was inhibited by the Nav1.7 antagonist Protoxin II (ProTx-II), and by tetrodotoxin (TTX), a nonselective sodium channel inhibitor (Fig. 1I). These results showed that the differentiated neurons include functional sodium channel-expressing neurons. Our results demonstrated that transient expression of NEUROG3 followed by treatment with reprogramming small-molecule inhibitors rapidly differentiates iPSCs into functional sensory neurons.

### Lytic gene expression, DNA synthesis, and virus production in iNGN3-derived differentiated neurons

**Fig 2:**
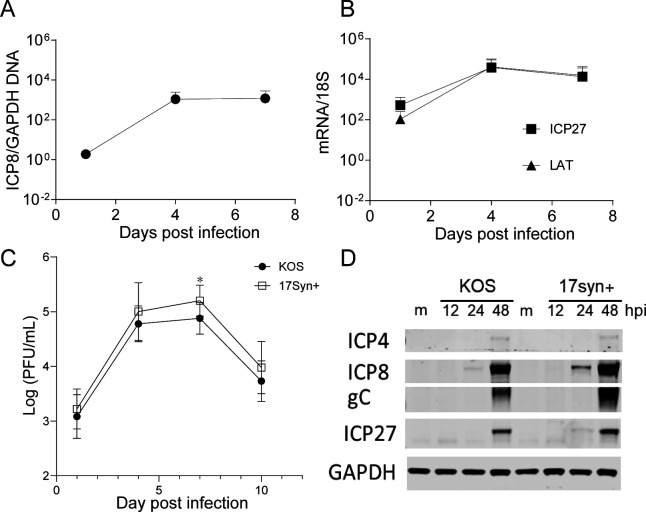
Lytic infection of HSV-1 in iNGN3-derived neurons. (**A and B**) iNGN3-derived neurons were infected with HSV-1 at an MOI of 1, and relative levels of vDNA and transcripts from *ICP27* and *LAT* genes were determined using qPCR and RT-qPCR. (**C and D**) iNGN3-derived neurons were infected with HSV-1 strain KOS or 17syn+ (MOI of 1), and viral titers were determined (**C**), and viral proteins were detected by Western blotting (**D**) using antibodies specific for infected cell protein 4 (ICP4), ICP27, ICP8, and gC at the indicated time points post-infection. GAPDH was detected as control. The graphs show the mean values and standard deviations of biological replicates, and statistical significance in (**C**) was evaluated by two-way ANOVA with Šídák’s multiple comparison tests for all four time points (*N* ≥ 3, *: *P* < 0.05).

To assess the kinetics of HSV-1 DNA synthesis, lytic gene expression, and virus production in these human neurons, we infected them (18–21 days differentiation) with wild-type (WT) HSV-1 strain KOS virus at a multiplicity of infection (MOI) of 1, harvested cells at various days post-infection (dpi), and analyzed levels of viral DNA (vDNA), transcripts, proteins, and infectious virus production. HSV-1 DNA increased by 10- to 100-fold by 4 dpi, and transcripts from the immediate–early (IE) *ICP27* gene and LAT transcripts also increased by 100-fold by 4 dpi (Fig. 2A and B). To examine the kinetics of viral replication, we harvested infected neurons at various dpi and assessed viral yields by plaque assays. Infectious HSV-1 strain KOS increased ~100 fold between 1 and 4 dpi and then plateaued before dropping between 7 and 10 dpi (Fig. 2C), which was similar to the kinetics of vDNA accumulation and consistent with earlier reports showing accumulation of infectious virus at 2 dpi (50, 51). We also tested the HSV-1 17syn + strain, which shows some differing properties from certain sub-strains of KOS (44). HSV-1 strains KOS and 17syn + showed similar kinetics of virus replication and about 100-fold increases in viral titer, but strain 17 showed slightly, but significantly higher levels of progeny virus (Fig. 2C). We also detected viral protein expression of KOS and 17syn + and found that levels of viral protein expression (ICP27, ICP8, and gC) of 17syn + were slightly higher than KOS (Fig. 2D) at the same hpi. These results showed that differentiated neurons support moderate HSV-1 lytic replication compared to Vero or HFF cells (52), and the 17syn + strain replicates somewhat more efficiently than the KOS strain in our neuronal system.

### iNGN3-derived differentiated neurons support establishment of latent HSV-1 infection

**Fig 3:**
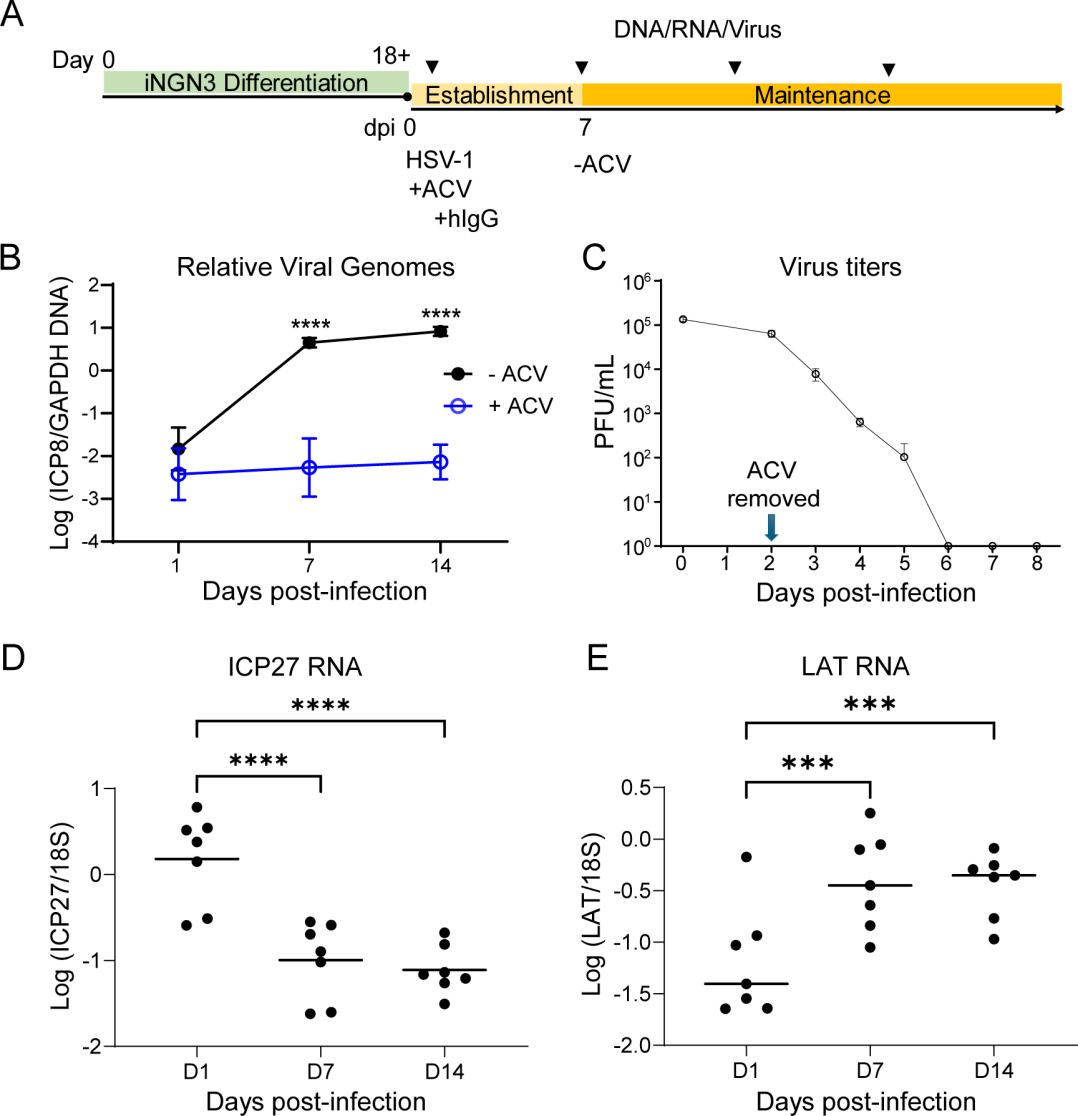
Evaluation of latent infection of HSV-1 in iNGN3-derived neurons. (**A**) Protocol and timeline for neuronal differentiation and HSV-1 infection. NGN3-derived neurons were infected with HSV-1 at an MOI of 1 in the presence of ACV. Human IgG (hIgG) was added to the media at 2 hpi and maintained for 7 days throughout the experiments, except in panel C where IgG was not used. ACV and hIgG were removed at 7 dpi in all experiments, except panel C. Cells were harvested to analyze virus, DNA, and RNA at various days post-infection (dpi). (**B**) For measurement of vDNA levels, iNGN3-derived neurons were infected with HSV-1 at an MOI of 1 in the absence or presence of ACV (200 µM). Latent HSV-1 infection was established for 7 days in the presence of ACV, and vDNA levels were measured at 1, 7, and 14 dpi. (**C**) For measurement of establishment of latent infection, we infected iNGN3-derived neurons with HSV-1 at an MOI of 1 in the presence of ACV (200 µM) and no hIgG. ACV was removed at 2 days. Infectious virus of the combined supernatant and cell lysates was monitored by plaque assay at the indicated dpi. (**D and E**) For measurement of lytic and latent transcripts, iNGN3-derived neurons were infected with HSV-1 at an MOI of 1 in the absence or presence of ACV (200 µM). Latent HSV-1 infection was established for 7 days in the presence of ACV, and lytic ICP27 gene transcripts (**D**), and LAT transcripts (**E**) were measured by RT-qPCR at the indicated dpi. The graphs show the mean values and standard deviations of biological replicates, and statistical significance was evaluated by two-way ANOVA of the logarithms of the plotted values with Šídák’s multiple comparison tests for all three time points for (**B**) and repeated measures one-way ANOVA with Dunnett’s tests to correct for multiple comparisons for (**D**) and (**E**) (*N* ≥ 3, ***: *P* < 0.001, and ****: *P* < 0.0001).

To examine whether HSV-1 could establish latent infection in the iPSC-derived sensory neurons, we infected differentiated neurons with HSV-1 strain KOS (MOI of 1) with or without the viral DNA polymerase inhibitor acyclovir (ACV, 200 µM) (Fig. 3A). Transient inhibition of viral replication by inhibitors is necessary to allow latent infection in most other cultured human and rodent neuron systems (27, 28, 43, 53). The viral inoculum was replaced at 2 hpi with neuronal culture media containing ACV and human immune globulin G (hIgG). We added hIgG to neutralize any residual HSV-1 to prevent potential superinfection and to minimize spreading of spontaneously reactivated HSV-1 in the latently infected cells. We replaced ACV- and hIgG-containing media every day and removed ACV and hIgG from the media starting from 7 dpi.

To monitor lytic versus latent infection, first, we measured vDNA levels at various dpi using qPCR. In the absence of ACV, the levels of vDNA increased from days 1 to 14 (Fig. 3B), while with ACV, HSV-1 DNA increased little if at all from days 1–14 (Fig. 3B). This difference is akin to the difference in ganglionic DNA levels during establishment of latent infection between KOS and a mutant that is replication-defective in mouse ganglia (54).

An important component of the basic definition of latent infection is the absence of infectious virus. To measure the decay of infectious virus during establishment of latent infection in these neurons, we infected the iPSC-derived neurons with WT KOS virus for 2 days in the presence of ACV (without hIgG for plaque assay), and then we titrated the infectious virus at various time points after ACV removal. We observed that infectious virus decreased substantially each day and was undetectable by day 6 (Fig. 3C). These results argued that latent infection was established in these cells by 6 dpi.

To further define the establishment of latency, we measured viral transcripts by RT-qPCR. In the presence of ACV, *ICP27* lytic gene transcripts decreased while *LAT* transcripts continued to increase during the ACV and hIgG treatment (up to 7 days) (Fig. 3D and E), which is qualitatively similar to the phenotype of viral transcripts during the establishment of latency of HSV-1 (for 28 dpi) *in vivo* (54). After removal of ACV and hIgG, lytic gene transcripts and LAT maintained constant levels (from 7 to 14 dpi). We measured the levels of *ICP27* transcripts at 21, 28, and 35 dpi and found that the levels were similar to or lower than the levels at 14 dpi (Fig. S2). These results indicated that iNGN3-derived neurons can support latent infection HSV-1 and showed similarities to the way lytic transcripts decrease and LAT transcripts increase in an *in vivo* mouse model.

### Expression of LAT during latency is dependent on its promoter

**Fig 4:**
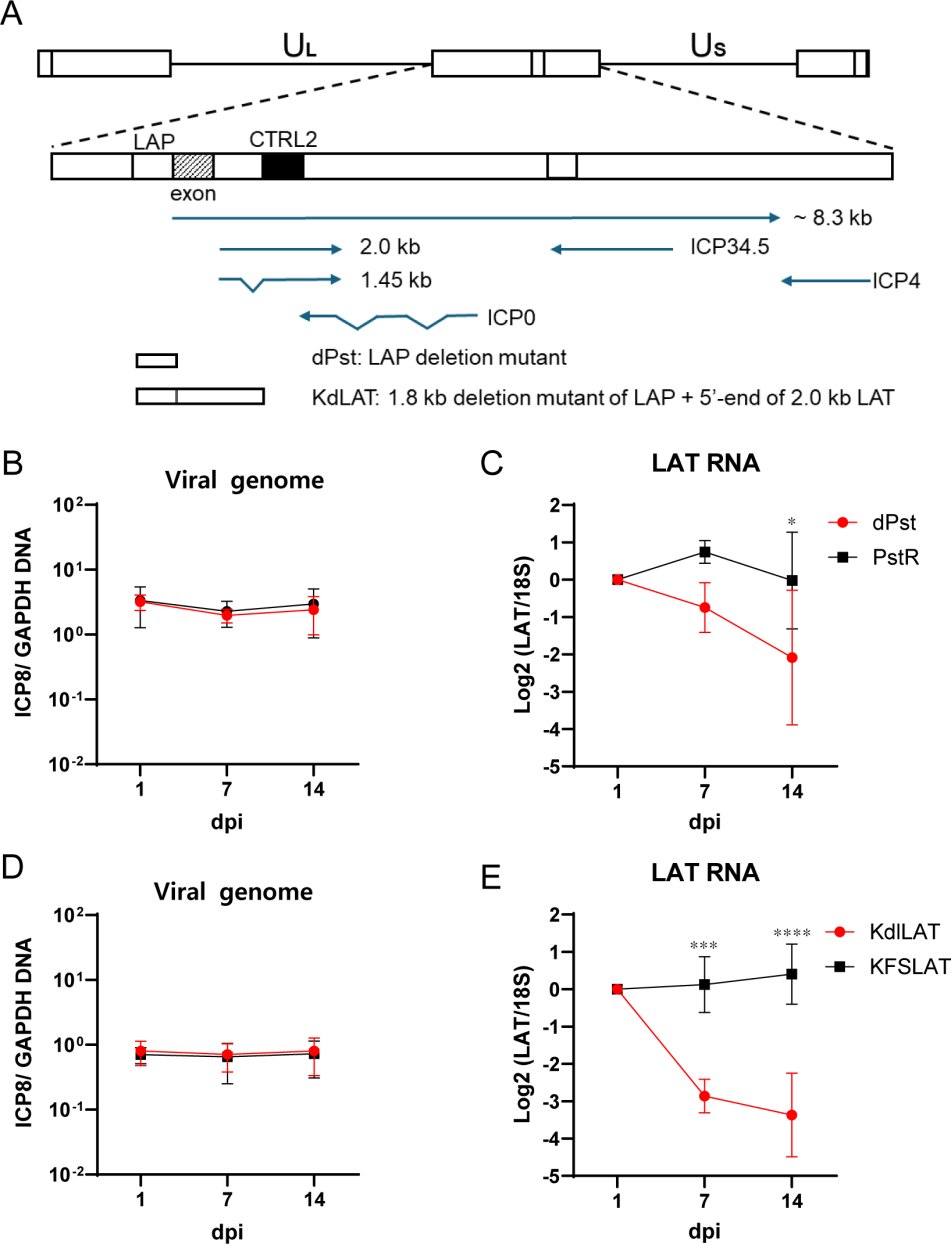
Viral genome and RNA levels of latently infected *LAT* mutants and their rescued viruses in iNGN3-derived neurons. (**A**) Schematic diagram of HSV-1 KOS K*dl*LAT and *d*Pst mutant genomes. LAP: LAT promoter (**B–E**) iNGN3-derived neurons were infected with the indicated HSV-1 strains at an MOI of 1 with ACV (200 µM) and hIgG for 7 days. (**B and C**) Relative levels of vDNA (**B**) and LAT (**C**) of latently infected *d*Pst and PstR viruses in differentiated neurons were determined by qPCR and RT-qPCR, respectively, at the indicated dpi. (**D and E**) Relative levels of vDNA (**D**) and LAT (**E**) of K*dl*LAT and KFSLAT were quantified by qPCR and RT-qPCR at the indicated dpi. Statistical significance at 7 and 14 dpi was evaluated by two-way ANOVA with Šídák’s multiple comparison tests for (**C**) and (**E**). *N* ≥ 4, *P < 0.05, ***: *P* < 0.001, and ****: *P* < 0.0001, and the error bars represent standard deviations.

Transcripts bearing *LAT* sequences can be derived from transcription from the authentic *LAT* promoter (*LAP*) during latent infection (55) or read-through transcription during lytic infection (56). To determine if the LAT transcripts detected in the latent infections of these neurons were expressed from *LAP*, we examined the levels of LAT with two *LAP* deletion mutant viruses in our differentiated sensory neurons. The HSV-1 KOS *d*Pst mutant virus contains a 202 bp deletion from the core of *LAP* (Fig. 4A), whose LAT transcript levels are about 1,000-fold decreased compared to its rescued virus PstR in TGs of latently infected mice (20). During latent infection in the differentiated neurons, *d*Pst and PstR showed equivalent levels of latent vDNA (Fig. 4B), and *d*Pst showed reduced levels of LAT by 14 dpi compared to its rescued virus PstR (Fig. 4C). A second mutant virus, K*dl*LAT, contains a 1.8-kbp deletion of the promoter, five exons, and part of the *LAT* intron region (Fig. 4A) (11, 57). Both K*dl*LAT and its rescued virus KFSLAT showed equivalent levels of latent vDNA (Fig. 4D). Interestingly, LAT transcript levels in K*dl*LAT-infected neurons were significantly decreased compared to its rescued virus KFSLAT on 7 dpi (Fig. 4E), which was sooner than the reduction of LAT of *d*Pst compared to PstR (14 dpi). In addition, the level of reduction of the LAT transcript for K*dl*LAT vs its rescued virus KFSLAT was greater (12-fold) than the difference of the LAT transcript between dPst vs PstR (4.6-fold). These results indicated that the authentic *LAT* promoter was indeed required for efficient LAT expression within these human sensory neurons.

### Latent HSV-1 DNA is associated with histones bearing heterochromatin markers

**Fig 5:**
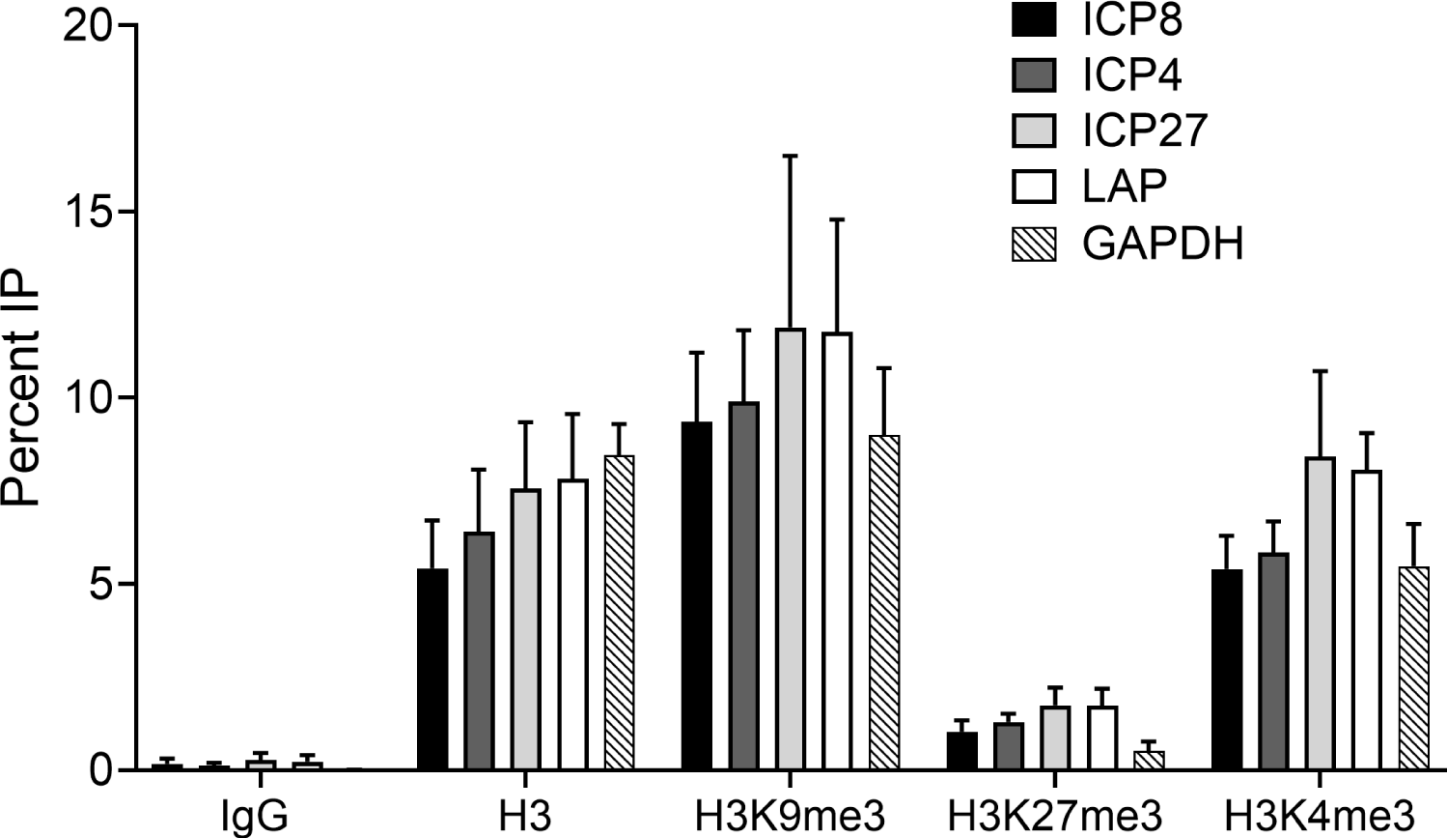
Bivalent chromatin on the HSV-1 genome during latent infection in iNGN3-derived neurons. Differentiated neurons were infected with HSV-1 (MOI of 1) in the presence of ACV and hIgG and harvested at 14 dpi for CHIP-qPCR analyses. Total histone H3, H3K9me3, H3K27me3, and H3K4me3 on viral (*ICP8, ICP4*, *ICP27*, and *LAP*) and host (*GAPDH*) promoters were analyzed by ChIP-qPCR.

To address the nature of the chromatin on the latent genomes in this system, we used chromatin immunoprecipitation (ChIP), as performed previously (20). Using ChIP assays, we found that histone H3 and heterochromatin markers H3K9me3 and H3K27me3 were associated with the *ICP4*, *ICP8*, and *ICP27* lytic gene promoters and *LAP* during latent infection at 14 dpi (Fig. 5). We also observed H3K4me3-modified histones on HSV-1 viral promoters of *ICP4*, *ICP27,* and *ICP8* and *LAP* at 14 dpi. Very low levels of DNA were immunoprecipitated with control IgG. These results argued that HSV-1 latent genomes are associated with heterochromatin and with H3K4me3, suggesting that they may be associated with bivalent chromatin. Single-genome studies are needed to confirm that euchromatic and heterochromatic markers are on the same viral genome.

### Latent HSV-1 in iNGN3-derived differentiated neurons can be reactivated by multiple stimuli

**Fig 6:**
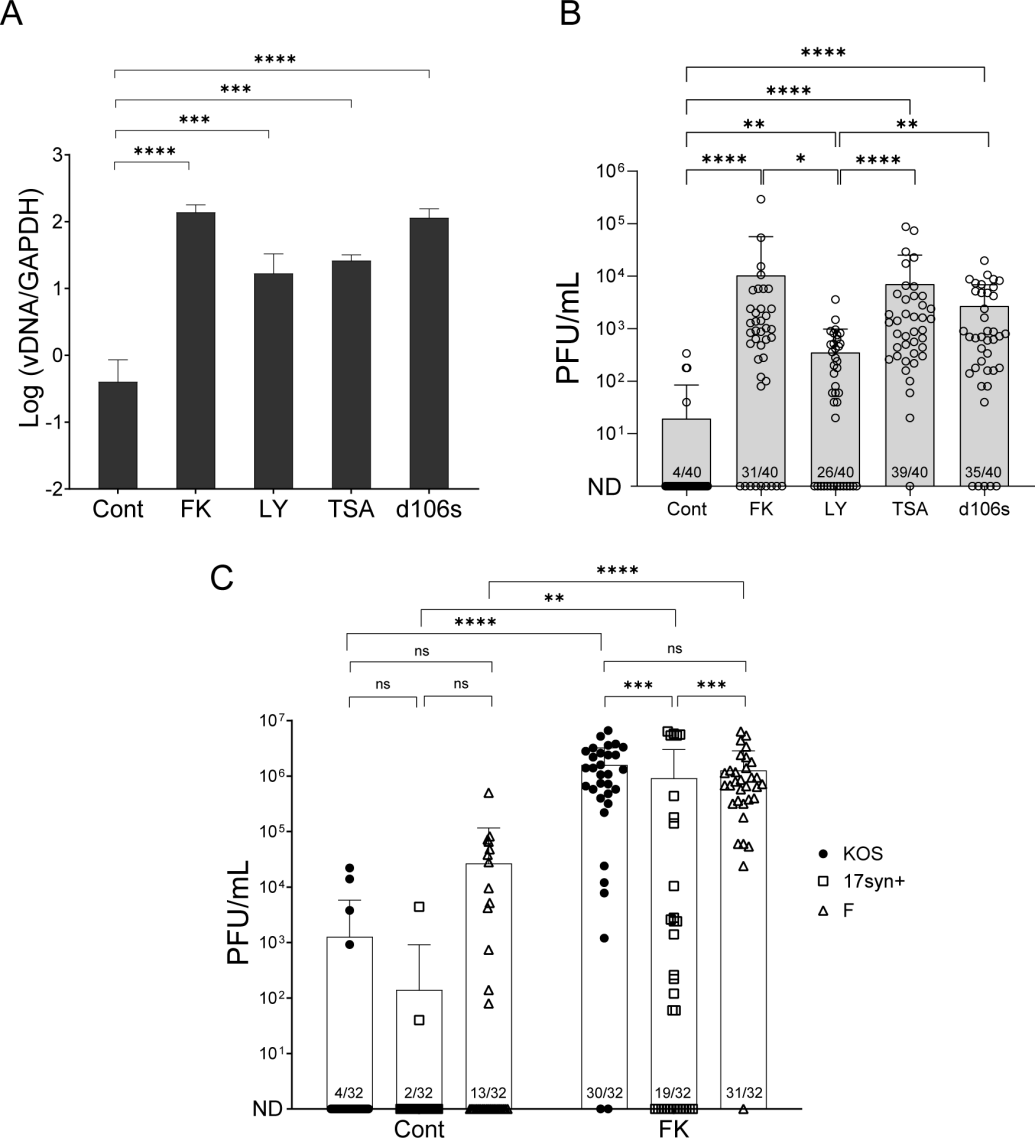
Induced reactivation of latent HSV-1. Differentiated neurons were infected with HSV-1 (MOI of 1) in the presence of ACV (200 µM) and hIgG, and reactivation was induced using the indicated stimuli at 1 day post-removal ACV and hIgG. (**A and B**) Relative vDNA and infectious virus levels were determined by qPCR (**A**) and plaque assay (**B**), respectively. Cont: DMSO-treated negative control, FK: forskolin (50 µM), LY: LY294002 (100 µM), TSA: (trichostatin A, 1.2 µM for vDNA and 1 µM for titers), *d*106s (MOI of 10). (**C**) Differentiated neurons were infected with KOS, 17syn+, or F strains of HSV-1 (MOI of 1) in the presence of ACV (200 µM) and hIgG for 7 days. ACV and hIgG were removed prior to reactivation induction. Individual stimuli were incubated for 3 days and replaced with fresh media. At 7 days post-induction of reactivation, the combined total cell lysates and supernatant were harvested, and infectious virus titer was determined by plaque assay. The numbers in each column represent the reactivated samples out of the total number of samples. The graph shows the mean values and standard deviations of biological replicates, with each symbol representing a different replicate. The fraction of replicates that reactivated is shown at the bottom of each bar. Statistical significance was evaluated by (**A**) one-way ANOVA with Dunnett’s test to correct for multiple comparisons, (**B**) and (**C**) Kruskal-Wallis test with Dunn’s test to correct for multiple comparisons (*N* ≥ 3, *: *P* < 0.05, **: *P* < 0.01, ***: *P* < 0.001, and ****: *P* < 0.0001), and the error bars represent the standard deviations. ND: not detected.

To examine the potential of the latent virus in these human sensory neurons to reactivate, a critical feature of authentic latent infection, we tested several stimuli known to reactivate HSV-1 in other neuronal cell models. We infected differentiated neurons with HSV-1 (KOS) at an MOI of 1 and established latent infection in the presence of ACV (200 µM) and hIgG for 7 d. At 1–2 days after ACV and hIgG removal, we tested for induction of reactivation of HSV-1 by the addition of various stimuli including forskolin (FK), PI3K inhibitor LY294002 (LY), or HDAC inhibitor TSA with DMSO as the vehicle control. We also superinfected cells with *d*106S virus, in which all the IE genes are deleted, except for *ICP0*, for transient expression of ICP0 (58). We harvested cells at 4 or 7 days after the addition of stimuli and determined viral genome levels by qPCR (Fig. 6A) or infectious virus by plaque assay (Fig. 6B), respectively. We detected low levels of spontaneous reactivation with DMSO, but we found that all the tested stimuli induced statistically significantly higher levels of reactivation compared to the vehicle control (Fig. 6A and B). The reactivation induced by the PI3K inhibitor LY was less efficient than that induced by other stimuli. These results argued that our sensory neuronal system maintains the signaling pathways involved in FK, LY, and TSA for HSV-1 reactivation shown in other neuronal models.

Previous studies found that different HSV-1 strains vary in their life cycle and reactivation potential (28, 44). To investigate how distinct HSV-1 strains behaved in our neuronal system during induced reactivation, we infected differentiated neurons with the KOS, 17syn+, or F strains of HSV-1 at an MOI of 1 and established latent infection for 7 days in the presence of ACV (200 µM) and hIgG. Reactivation was induced using FK, revealing that the KOS and F strains displayed similar reactivation frequencies and levels of infectious virus. Notably, the 17syn + strain exhibited significantly lower induced reactivation compared to the other two strains (Fig. 6C). In addition, the F strain virus showed more frequent spontaneous reactivation than the other two strains, although it is not statistically significant.

In summary, we found that HSV-1 establishes latent infections in iNGN3-derived sensory neurons, which can be reactivated by various stimuli. Furthermore, distinct HSV-1 strains show varying sensitivities to FK-induced reactivation. These results highlight the utility of our iNGN3-derived sensory neuron-enriched culture model as a valuable tool for studying the molecular and biological mechanisms underlying HSV-1 lytic infection, latency, and reactivation under diverse conditions and stimuli.

## DISCUSSION

HSV-1 establishes latent infection in human sensory neurons and persists for the life of the individual. Many of the experimental studies of HSV-1 latency have been conducted in animal model systems because human sensory neurons have not been available in sufficient quantities to conduct molecular biology studies. To address this need, we have used iNGN3 iPSCs to differentiate sensory neurons and validated them by gene expression and physiological studies. The iNGN3-derived neurons supported both lytic and latent HSV-1 infections. Latent infection was validated by demonstration of the lack of infectious virus, expression of LAT dependent on its authentic promoter, repression of lytic gene expression, persistence of vDNA associated with heterochromatin, and the ability to reactivate from latency with appropriate stimuli.

### Validation of iNGN3-derived neurons as sensory neurons

The iNGN3 iPSCs that we used encode a Dox-inducible NEUROG3 gene that drives differentiation into diverse subtypes of functional bipolar neurons within 4 days (46). Single or combined NGN family induction differentiates iPSCs into heterogeneous neurons (59–62). We further differentiated them into sensory neurons using small molecules, a component of the “Chambers method” (47–49). However, the prior approaches require extended time frames and cell sorting to enrich sensory neuron populations, often resulting in significant cell loss (47). In contrast, our approach combining NGN3 induction with small-molecule differentiators generates a high yield of sensory neurons in less than 2–3 weeks without the need for sorting. This makes the approach more scalable and flexible than the Chambers method. Additionally, this strategy of combining small molecules with transcription factor induction offers a potential approach to accelerate the differentiation of stem cells into other neuronal subtypes.

TGs and DRGs harbor various sensory neuronal subtypes. i) nociceptors: noxious stimulus sensitive (TRKA, TAC1, and CGRP), ii) mechanoreceptors: touch and pressure (TRKB and CNTNAP2), iii) mechanosensitive proprioceptors: body position and movement (TRKC and parvalbumin), iv) thermoreceptors: temperature changes (TRPV1 and TRPM8), 5) pruriceptors: itch stimuli (MrgprA3 and TRPV1). The iNGN3-derived neurons show robust expressions of *TrkA*, *TrkB*, and *TrkC*, which are hallmark markers for nociceptors, mechanoreceptors, and proprioceptors, respectively. Notably, while *RUNX1*, a transcription factor known for *TrkA* upregulation in nociceptor neurons (63, 64), was not detected in our RNA-sequencing data, alternative transcription factors such as *Klf7* and *Brn3a*, which are also reported to promote *TrkA* expression in sensory neurons, were highly expressed (65, 66).

iNGN3-derived neurons had low levels of *TRPV1* expression; however, capsaicin stimulation successfully induced a calcium influx, confirming the functionality of *TRPV1* in these neurons. Interestingly, while the *TRPA1* transcript was barely detectable, its agonist, allyl isothiocyanate (AITC), still induced calcium influx. Although *TRPA1* is recognized as the primary receptor for AITC sensing, high concentrations of AITC can activate *TRPV1* (67, 68). Therefore, the observed AITC-induced calcium influx may primarily result from *TRPV1* activity. It is also possible that other, as-yet unidentified receptors may contribute to AITC sensitivity. These alternative receptors could be screened and identified using our neuronal model.

In addition to transient receptor potential (TRP) channels, voltage-gated sodium (NaV) channels are essential for initiating and conducting action potentials in sensory neurons. Most ESC- and iPSC-derived neurons typically require extended differentiation for NaV channel expression (47, 69). Our system enables functional NaV channel expression in less than 3 weeks, although it remains to be determined whether these differentiated neurons are fully mature. The functionality of our sensory neurons is similar to that of other human iPSC-derived sensory neurons (45).

Human and mouse trigeminal ganglia (TGs) comprise 15 transcriptionally distinct subtypes of neurons (40, 42). While they share 86.8% of their genes as either equally present or absent across species, differences exist in the gene expression profiles of their neuronal subtypes. For example, genes such as CALCA, CALCB, DPP10, HTR3A, LRRC4C, FSTL, FNDC5, LPAR3, CACNB2, and BBS9 are highly expressed in human neuron subtypes but are absent in mouse counterparts. Interestingly, except for CALCA and CALCB, most of these genes are also highly expressed in our iNGN3-derived neurons, suggesting that they capture characteristics of specific subtypes of human sensory neurons. Single-cell analysis can determine populations of more specific neuronal subtypes in our iNGN3-derived neurons. This highlights the potential of this approach to more accurately reflect human sensory neuronal biology, particularly in the context of HSV-1 studies.

### Lytic and latent infection

In the absence of acyclovir, the iPSC-derived sensory neurons undergo a limited lytic infection with genome amplification of 10-100-fold, and infectious virus increases of 100-fold, less than more permissive fibroblast cells (70). Nevertheless, the addition of the antiviral acyclovir was needed to reduce the likelihood of lytic infection to allow establishment of a latent infection. Antiviral treatment is needed for latent infection in nearly all cell-based infection models with wild-type virus, except at low MOI or infection by the axonal route in multi-chamber culture systems (71). In the presence of ACV, vDNA levels are constant over the times tested, 1–21 dpi, consistent with a latent infection.

In our system, LAT expression increased as latent infection was established, while the lytic *ICP27* gene transcript decreased. LAT levels were reduced by 10–100-fold in our cells by deletion of *LAP*. Larger decreases in transcripts with the promoter deletion mutants occurred in latently infected murine ganglia, e.g., 10^3^–10^6^-fold with KOS*d*PstLAT (11, 20). This could mean that LAT transcription is lower in these neurons at the time points examined, or that there is more read-through from other promoters in the human neurons. Later time points post-latent infection in the iPSC-derived neurons may show higher levels of LAT accumulation. In contrast, HD10.6 DGR neurons and LUHMES latently infected neurons showed decreasing LAT levels as latent infection is established (28, 43). Thus, our cells may be unique for human neuronal cells tested thus far in showing increased levels of LAT as latent infection is established.

### Viral Chromatin

Lytic genes on latent HSV-1 DNA are associated with facultative heterochromatin in mouse ganglia including H3K9me2, H3K9me3, and H3K27me3 (19–21). The iNGN3-derived sensory neurons showed similar heterochromatin markers on latent HSV-1 DNA but also showed the euchromatin marker H3K4me3. The presence of both H3K27me3 and H3K4me3 modifications may indicate bivalent chromatin, which is found on developmentally regulated genes and provides epigenetic silencing that can be readily reversed for developmental processes (72, 73). Thus, bivalent chromatin would be ideal for maintenance of latent infection in a form that is “poised” for reactivation (74). However, it has not been confirmed that an individual genome harbors both markers. Single-genome resolution studies are required to determine the co-existence of both markers on the same genomes.

### Reactivation

The ability to reactivate from latent infection is an essential component of authentic latent infection. Animal models have provided much of our experimental knowledge of this process, but it is important to determine the mechanisms of reactivation in human sensory neurons. In our studies, we observed that various stimuli could induce HSV reactivation in iNGN3-derived neurons, supporting the possible involvement of multiple pathways. Furthermore, these results reinforce studies using animal models and neurons derived from animals.

Previous studies have found that different types of neurons vary in their responses to various stimuli. For example, reactivation of HSV using neurons from TGs and SCGs differs for reactivation induced by epinephrine or corticosterone (75). Neurons differentiated from HD10.6 cells exhibit limited reactivation in response to a range of stimuli (28). Because these neuronal populations consist of varying proportions of distinct subtypes, it is possible that specific stimuli selectively trigger reactivation in particular neuronal subtypes based on their unique gene expression profiles. iNGN3-derived neurons appeared to exhibit a lower frequency of reactivation with a PI3K inhibitor than FK or TSA, indicating that a PI3K involved pathway might be less active than the FK triggering pathway in these neurons. A reactivation assay at the single-cell level could uncover specific pathways, providing deeper insights into the mechanisms governing HSV-1 reactivation in distinct neuronal subtypes.

Different strains of HSV-1 demonstrated the ability to reactivate in iNGN3-derived neurons, with notable variations in their behavior. The more neurovirulent strain 17syn + exhibited more robust lytic replication compared to the KOS strain. However, the KOS strain showed a more vigorous reactivation than the 17syn + strain. In addition, the low-passage F strain appeared to exhibit a higher frequency of spontaneous reactivation compared to both the KOS and 17syn + strains, suggesting that strain-specific factors influence latent infection and reactivation dynamics. A recent study reported that 17syn + produced slightly higher levels of lytic and *LAT* gene transcripts and reactivation compared to KOS in LUHMES-matured neurons (44). However, the “KOS” strain used in that work was the KOS1.1 sub-strain previously shown to be reduced for latent infection (76). Further studies into the chromatin profiles of different HSV-1 strains during latent infection are necessary to elucidate the mechanisms underlying these strain-specific differences.

In summary, we have developed a human sensory neuronal model that will serve as a valuable tool for studying HSV-1 latency and reactivation in human sensory neurons. This model enables detailed exploration of the molecular and biological mechanisms underlying these processes and facilitates the identification of potential therapeutic targets for preventing or treating HSV-1 reactivation.

## MATERIALS AND METHODS

### Cells and viruses

NEUROG3 iPSCs (iNGN3) were derived from the Personal Genome Project Participant 1 (PGP1) iPSCs by introduction of a Dox-inducible neurogenin 3 gene (46). Vero cells were obtained from the American Type Culture Collection. Vero cells were maintained as described previously (70). HFF cells were maintained in DMEM supplemented with 10% (vol/vol) FBS (77). Vero cells were used to grow and titrate the HSV-1 KOS wild-type (WT) strain (70), *d*Pst, PstR (20), K*d*LAT, and KFSLAT (57). E11 cells were used to grow and titrate HSV-1 *d*106 (58) provided by Neal DeLuca, University of Pittsburgh.

### Neuronal cell differentiation

iNGN3 cells were seeded in Matrigel (STEMCELL)-coated 12-well plates (Corning or Costar, 5 × 10^4^ cells), 6-well plates (Corning or Costar, 1 × 10^6^ cells for replating), or 100 mm dish (Corning, 4 × 10^6^ cells) and induced with doxycycline (Dox, 1µg/mL) containing mTeSR +supplement (STEMCELL) for 3 days. The medium was then changed to mTeSR/N2 media (mTeSR +supplement: Neurobasal plus +N2 supplement (Thermo Fisher Scientific) =1:1) with five inhibitors (5i; LDN193189 (APExBIO, 0.1 µM), SB431542 (APExBIO, 10 µM), DAPT (Cayman Chemical or APExBIO, 10 µM), SU5402 (Cayman Chemical or APExBIO, 10 µM), and CHIR99021 (Sigma or APExBIO, 5 µM)) with daily changes for 5 days. After 5i treatment, cells were maintained in the Matrigel-coated plate or re-plated to poly-L-lysine- (PDL, 50–100 µg/mL, Sigma)/laminin- (10 µg/mL, Gibco) or poly-L-ornithine- (POL, 50–100 µg/mL, Sigma)/laminin- (10 µg/mL, Gibco) coated plates. To promote neuronal maturation, the cells were further differentiated in 4G media, which contained four growth factors (4G; BDNF (PeproTech, 10 ng/mL), GDNF (PeproTech, 10 ng/mL), β-NGF (PeproTech, 10 ng/mL), and NT-3 (PeproTech, 10 ng/m;)), ascorbic acid (0.2 mM) in B27 media (Neurobasal plus +B27 plus (Thermo Fisher Scientific):DMEM/F12 (Thermo Fisher Scientific) =1:1)). To eliminate undifferentiated dividing cells, mitomycin C (MMC, STEMCELL) or 5-fluoro-2'-deoxyuridine (20 µM, FUdR, RPI) was used. For MMC treatment, MMC (10 µg/mL) was incubated with the cells for 2.25 h at 37°C, washed twice with DMEM/F12, and maintained with 4G media at 37°C. For FUdR treatment, FUdR (20 µM) was added to 4G media and maintained for 7 days. The cells were maintained in 4G media with media changes every 2–3 days.

### Immunofluorescence microscopy

Immunofluorescence imaging was performed as described previously (52). Briefly, iNGN3 cells (25,000/well) were seeded on Matrigel-coated coverslips and differentiated as described above. The differentiated cells were replaced on poly-L-ornithine (POL, 50–100 µg/mL, Sigma)/laminin (10 µg/mL, Gibco) coated sterile coverslips (FisherBrand; 12-545-81) or 96-well black with clear flat-bottom plate (CORNING 3340). The cells were fixed with 3.7% paraformaldehyde in PBS for 10 min at room temperature, permeabilized with 0.1% Triton X-100 in PBS for 10 min, and incubated in 5%–10% normal goat serum (Jackson ImmunoResearch Laboratories; 005-000-121)-containing blocking buffer for 1 h at room temperature or overnight at 4°C. The cells were incubated with the primary antibodies (Sox2 (Abcam, ab97959, 1:500), BRN3a (Millipore, MAB1585, 1:500-700), ISL1 (DSHB, 40.2D6, 1:50 or Abcam, ab20670, 1:500), and NeuN (Millipore, MAB 377, 1:100), NAV 1.7 (Abcam, 1:500, ab65167), TRPV1 (VR1) (Alomone Labs, 1:500), TRKA (Thermo Fisher Scientific, MA5-32123, 1:300), TRKC (Thermo Fisher Scientific, 7H3L20, 1:300), peripherin (Millipore, MAB1527, 1:700), anti-B-III tubulin (Millipore, MAB1637, 1:700) for 1 h at room temperature or overnight at 4°C and then washed with 0.1% Tween 20 in PBS three times. Secondary antibodies conjugated to Alexa 594, Alexa 647, and 488 dyes (Life Technologies, 1:500) were subsequently used for 1 h at room temperature. Nuclei were stained with 2-(4-amidinophenyl)−1*H*-indole-6-carboxamidine (DAPI, Life Technologies), and the coverslips were mounted using Prolong Gold Antifade mounting reagent (Life Technologies). Images were acquired with either a Zeiss Axioplan 2 microscope with a Plan Apochromat 63 × 1.4 N.A. objective lens, a Photometrics CoolSNAP HQ2 CCD camera, and processed with the Zeiss Axiovision 4.8 image acquisition software or a Nikon Ti Inverted Microscope and Nikon Elements 4.30 Acquisition Software. Images were processed using Fiji or Axiovision 4.8. For higher-magnification images, iPSC and neurites were seeded on sterile coverslips (FisherBrand; 12-545-81) using 24-well plates (Corning, 3524). Cells were fixed in 3.7% (vol/vol) formaldehyde solution diluted in PBS (Sigma, F8875) for 10 minutes at room temperature. After discarding formaldehyde through three washing steps in PBS, cells were permeabilized with 0.1% (vol/vol) Triton X-100 solution made in PBS for 10 minutes at room temperature. Cells were blocked for 1 h at 37°C using 5% (vol/vol) normal goat serum (NGS; Jackson ImmunoResearch Laboratories; 005-000-121), 0.16% (vol/vol) human immunoglobulin infusion (Gammagard 10% Liquid, Cencora, 00944-2700-04), and 1% (vol/vol) heat-inactivated BCS, all diluted in DMEM. Primary antibodies were diluted in 5% (vol/vol) NGS diluted in 0.2 μm-filtered PBS and incubated overnight at 4°C. The primary antibodies used were anti-Nav1.7 antibody (Abcam, ab65167, 1:500 dilution), anti-Tuj1 antibody (Millipore, AB9354, 1:200 dilution), anti-peripherin antibody (Millipore, MAB1527, 1:500 dilution), anti-TRPV1 antibody (Alomone Labs, ACC-030, 1:200 dilution), anti-Brn3a antibody (Millipore, MAB1585, 1:500 dilution), anti-TrkB antibody (Protein Tech, 13129-1-AP, 1:25 dilution), anti-TrkC antibody (ThermoFisher, 7H3L20, 1:300 dilution), anti-Islet1 antibody (Developmental Studies Hybridoma Bank, 40.2D6, 1:50 dilution), anti-NeuN antibody (Millipore, MA377, 1:100 dilution), anti-Nav1.8 antibody (Alomone Labs, SCN10A, 1:100 dilution), anti-TRPA1 antibody (Alomone Labs, ACC-037, 1:50 dilution), anti-TRPM8 antibody (Alomone Labs, ACC-045, 1:100 dilution), and anti-SOX2 antibody (Abcam, ab97959, 1:200 dilution). The day after, the unbound primary antibodies were washed away with filtered PBS three times. A 1:500 dilution of the appropriate fluorochrome-conjugated antibodies (Invitrogen, A21236, A11034, and 11042) was added to fixed cell monolayers for 1 h at 37°C, diluted in 5% NGS solution along with 4',6-diamidino-2-phenylindole, dilactate (DAPI, ThermoFisher, D3571) for cell nuclei counterstaining. After three washing steps with filtered PBS, coverslips were mounted onto microscopy slides (VWR North American, 48311-703) using 3 µL drops of SlowFade Diamond Antifade Mountant (Invitrogen, S36967), and sealed with transparent nail polish. Confocal micrographs were taken at the Microscopy Resources on the North Quad of the Harvard Medical School (MicRoN) using a Nikon Ti inverted confocal microscope with a W1 Yokogawa Spinning disk (50 µm pinhole disk), with an Andor Zyla 4.2 Plus sCMOS monochrome camera through the Nikon Elements Acquisition Software AR 5.02 and a 100 x objective lens Plan Apo λD 100 x/1.45 Oil DIC using immersion oil type 37 (Cargille laboratories; 16909-04). The resulting images, taken with a 0.4 µm Z-stack width, were analyzed using ImageJ Version 2.14.0 (NIH, USA) and presented as Z-projections for the simultaneous visualization of neuronal cell bodies and neurites.

### Voltage clamp recording

Whole-cell patch-clamp recordings were performed using a Multiclamp 700B amplifier (Molecular Devices, Sunnyvale, CA, USA). Series resistance was compensated (>80%) with amplifier circuitry. Data were acquired at 50 kHz and filtered at 2 kHz. Electrodes (1.8–3.0 MΩ) were filled with pipette solution (140 mM CsF, 10 mM NaCl, 1.1 mM EGTA, 10 mM HEPES, 20 mM dextrose); pH was adjusted to 7.3 with CsOH, and the osmolality was adjusted to 280–290 mOsm with sucrose. The bath solution: 30 mM NaCl, 90 mM choline-Cl, 20 mM tetraethylammonium chloride (TEA-Cl), 3 mM KCl, 1 mM CaCl2, 1 mM MgCl2, 0.1 mM CdCl, 10 mM Hepes, and 10 mM dextrose; pH was adjusted to 7.4 with NaOH, and the osmolality was adjusted to 300–310 mOsm with sucrose. Neurons were held at −120 mV. Voltage-gated Na currents were evoked with a series of 100 ms command potentials from −120 to 0 mV. Leakage currents were digitally subtracted using a *P*/–4 leak subtraction protocol from a holding potential of −120 mV. 5 nM Protoxin-ii (Peptide international) and 300 nM TTX (Tocris) were used to isolate Nav1.7 current and TTX-S current, respectively.

### AmpliSeq

Neurons collected for transcriptomic analysis were lysed, and total RNA was isolated (Clontech, 740971.50). To prepare libraries for AmpliSeq whole-transcriptome analysis, the total RNA of each sample was diluted to 0.95 ng/µL, and 10.5 µL from each sample was loaded in each reverse transcription reaction with the SuperScript VILO cDNA Synthesis Kit (ThermoFisher, 11754050) and run in a thermocycler with the following settings: 42°C for 30 minutes, 85°C for 5 minutes, and 10°C hold. After reverse transcription, the libraries were prepped using the Ion Chef Robotic System (ThermoFisher, 4484177) and the Ion AmpliSeq Transcriptome Human Gene Expression Panel, Chef-Ready Kit (ThermoFisher, A31446), which allows for preparation and barcoding of eight libraries simultaneously. The AmpliSeq Human Gene Expression Panel results in amplification of one unique 100 bp amplicon from each transcript in the panel, and completed libraries from eight samples were pooled together, templated, and loaded on an Ion 540 Chip (ThermoFisher, A27765) using the Ion Chef Robotic System and the Ion 540 Kit Chef (ThermoFisher, A27759). Each 540 Chip was then inserted into the Ion Torrent S5 sequencer (ThermoFisher, A27212), and 500 nucleotide flows were applied. Mapping of reads to amplicons and thus individual genes was completed with the AmpliSeq Plugin available on the ThermoFisher S5 Torrent server. CHP files resulting from mapping of reads were loaded into the Transcriptome Analysis Console (TAC) software from ThermoFisher to complete differential gene expression analysis, with a 1.2-fold change in expression as cutoff and *P*-values corrected for multiple comparisons using False Discovery Rate (FDR).

### Calcium image staining

NGN3 neurons were loaded with 1 mM calcium indicator fura-2 AM ester (F1221, Invitrogen, 1 mg/mL) by diluting it to 4 µM in the media and incubating for 45 min at room temperature in the dark. After 45 min, cells were washed twice with standard external solution (SES) (145 mM NaCl, 5 mM KCl, 2 mM CaCl_2_, 1 mM MgCl_2_, 10 mM HEPES, 10 mM glucose, and pH 7.4). Cells were placed in a recording chamber of a Ti Eclipse inverted microscope (Nikon) and were continuously perfused with SES using ValveBank 8 II (AutoMate Scientific). The ratio (R) of fluorescence emission (510 nm) was measured in response to 340/380 nm excitation with an exposure time of 600 and 300 ms, respectively, using NIS-Element imaging software (Nikon). The change in fluorescence was normalized to the 30 s baseline fluorescence prior to the stimulations. Only active cells with a minimum 10% fura-2 AM ratio change from baseline during the last 40 mM KCl stimulation were included in the further analyses.

### HSV infection, establishment of latency, and reactivation

At 18–21 days post-differentiation, the iPSC-derived neuronal cells were infected with HSV-1 strains KOS, F, or 17syn + for 2 h, and acyclovir (ACV, Sigma, 200 µM) and hIgG (Takeda, Immune Globulin Infusion (human) 10% GAMMAGARDLIQUID, Cencora, 00944-2700-04, 0.16%) were added for establishment of latent infection. Media containing ACV alone or ACV and hIgG were replaced daily and removed at 7 dpi. Cells were harvested at the indicated time points to analyze vDNA and lytic gene and *LAT* transcripts. As a control, cells were infected with HSV without ACV treatment. For lytic infection, infected cells were incubated in the absence of ACV and harvested at the indicated time points. Reactivation was induced by addition of stimuli (FK: forskolin (APExBIO, 50 µM), LY: LY294002 (Cayman Chemical, 100 µM), TSA: (trichostatin A, APExBIO, 1.2 µM for vDNA and 1 µM for titers), and *d*106S virus (MOI 10) for 3 days, and cells were harvested to analyze viral genome (qPCR) and infectious virus (plaque assay) at the indicated time points. The virus titer was determined using the total cell lysate and culture supernatant together.

### Reverse transcription and real-time PCR

Reverse transcription and real-time PCR were performed as described previously (52). Briefly, DNA and RNA were purified and quantified using AllPrep DNA/RNA (Qiagen) according to the manufacturer’s protocol. An aliquot (0.5–1 μg) of the purified total RNA was treated with DNase I (Ambion) and reverse-transcribed using the high-capacity cDNA reverse transcription kit (Applied Biosystems). Relative amounts of specific cDNA in the reverse-transcribed cDNA and purified total DNA were quantified using their specific primers with the SYBR Green PCR Master Mix reagent (Applied Biosystems) and the StepOnePlus Real-Time PCR system (Life Technologies). Primers (IDT) used in this study are as follows: *GAPDH* (5′-TTCGACAGTCAGCCGCATCTTCTT-3′ and 5′-CAGGCGCCCAATACGACCAAATC-3′ (78)), *ICP8* (5′-GTCGTTACCGAGGGCTTCAA-3′ and 5′-GTTACCTTGTCCGAGCCTCC-3′), *18S* rRNA (5′-GTAACCCGTTGAACCCCATT-3′ and 5′-CCATCCAATCGGTAGTAGCG-3′), LAT (5′-TGTGTGGTGCCCGTGTCTT-3′ and 5′-CCAGCCAATCCGTGTCGG-3′), hNANOG (5′-TCTCGTATTTGCTGCATCGTA-3′ and 5′-AGCTAATTTCCTTCTCCACCCC-3′), hOCT4 (5′-CCCTTCGCAAGCCCTCATTTC-3′ and 5′-GGGCGAGAAGGCGAAATCC-3′), hBRN3 (5′-CCTGAGCACAAGTACCCGTC-3′ and 5′-CGGCTTGAAAGGATGGCTCT-3′), hSIX1 (5′-TACGCGCACAATCCCTACC-3′ and 5′-GGTCTCTTTGCCTCCGGTT-3′), hPRPH (5′-CATTGAGACCCGGAATGGG-3′ and 5′-GTGGGCAGAAGACTTGTCCA-3′), hTRKA (5′-CAACAACGGCAACTACACGC-3′ and 5′-TGCTGTTAGTGTCAGGGATGG-3′), hTRKB (5′-GCAAGAGCTGAACCAAGCAC-3′ and 5′-CCCGGTCCCTAATTCACACC-3′), hTRKC (5′-GCGTTTCAAAGAAGCAGCGA-3′ and 5′-CCAGCAAGAAAATCCGCCAG-3′), hP2R × 3 (5′-CTCCAACCCCTCTAAGCTGC-3′ and 5′-GCTCAGGGGACACTCAGAAG-3′), hTRPM8 (5′-ATGACACTCTGGACAGCACC-3′ and 5′-TCTGGGCATAGCCACACTTG-3′), hTRPA1 (5′-GTTTATTCCCTCACTACCCC-3′ and 5′-CAAGGACACATACATAGCCAA-3′), and hTRPV1 (5′-CAGAGTCACGCTGGCAACC-3′ and 5′-GAGACTCTCCATCACACTGTC-3′). To determine the relative levels of individual target genes, serial dilutions of purified total cellular and vDNA were used to generate standard curves for each gene. Only qPCR data that fell within the established standard curves were considered for analysis.

### Immunoblotting

Immunoblotting was performed as described previously (79, 80). Briefly, neurons were lysed in lysis buffer (31.25 mM Tris-HCL, pH 6.8, 10%–12.5% glycerol, 1% SDS, and 0.04%–0.05% Orange G) or in protein loading buffer (LI-COR: 928-4004) supplemented with protease inhibitors (cOmplete, EDTA-free, Roche; 05056489001). The proteins were resolved in NuPAGE 4%–12% Bis-Tris Gels (Life Technologies) and transferred to a nitrocellulose membrane (Bio-Rad, #1620112). The membranes were blocked in Odyssey Blocking Buffer (LI-COR) and incubated with antibodies specific for HSV-1 ICP8 (1:5,000, rabbit serum 3-83 (81), ICP27 (1:5,000, mAb, Eastcoast Bio), HSV-1 ICP4 (1:2,000, monoclonal mouse (mAb) 58S, purified from hybridoma cell line 58S (ATCC HB8183), or GAPDH (1:10,000, mAb, Abcam). The membranes were incubated with secondary antibodies, IRDye 680RD or IRDye 800 (LI-COR), for 45–60 min and imaged using Odyssey (LI-COR).

### Chromatin immunoprecipitation (ChIP) assay

ChIP analyses were performed as described previously (70, 82). Differentiated neurons were infected by KOS-1 and/or mutant viruses at an MOI of 1, with 200 µM ACV and hIgG (0.16%) for 7 days. ACV and hIgG were removed and maintained in 4G media. At indicated time points post-infection, cells were washed by serum-free medium and fixed with 1% formaldehyde at 37°C for 15 min. The formaldehyde crosslinking was quenched by 125 mM cold glycine and washed with PBS three times. Cells resuspended in PBS were sonicated at 4°C for three cycles of 5 min each in a Diagenode Bioruptor, on the high setting with 15 s on, 45 s off to shear the DNA. Equal amounts of chromatin were incubated with 2.5 µg of histone-specific antibodies (H3, Abcam ab1791; H3K9me3, Abcam ab8898; H3K27me3, Active Motif 39156; H3K4me3, Cell Signaling #9751; H3K18Ac, Abcam ab1191) or control IgG (Millipore 12-370 and 12-371) at 4°C overnight; 10% of each sample was reserved as the input. The immunocomplexes were pulled down through mixing with protein A magnetic beads (Magna ChIP, Millipore, 16-661) and later washed three times with cold, low-salt buffers (150 mM NaCl; 20 mM Tris-HCl; pH 8.1; 2 mM EDTA; 1% Triton X-100; 0.1% SDS; 1 mM PMSF), three times with cold, LiCl buffer (50 mM HEPES, pH 7.5; 250 mM LiCl; 1 mM EDTA; 1% NP-40; 0.7% sodium deoxycholate; 1 mM PMSF), and finally once with cold, Tris-EDTA buffer (10 mM Tris-HCl, pH 8; 1 mM EDTA). Samples were eluted with elution buffer (100 mM NaHCO3; 1% SDS,) and NaCl added to a final concentration of 200 mM and incubated overnight at 65°C to reverse the protein-DNA crosslink, followed by RNase A (Ambion) and proteinase K (Roche) treatment. DNA was purified using a QIAquick PCR purification kit (Qiagen) and then analyzed by quantitative-PCR using primers listed as below (20, 82). ICP4 F: GCGCTCCGTGTGGACGAT; ICP4 R: CGGCCCCTGGGACTATATGA. ICP8 promoter F: GAGACCGGGGTTGGGGAATGAATC; R: CCCCGGGGGTTGTCTGTGAAGG. ICP27 promoter F: ACCCAGCCAGCGTATCCACC; R: ACACCATAAGTACGTGGCATGT. LAT promoter F: CCCGGCCCGCACGAT; R: CAACACCCCGCCGCTTT. GAPDH promoter F: TACTAGCGGTTTTACGGGCG; R: TCGAACAGGAGGAGCAGAGAGCG. Tubulin beta-III promoter F: CGGCAAAAGCTCAGAGCAC; R: CTCGGTTCCGCCCTTGCG.

### Statistical analyses

Independent biological replicates are analyzed to determine the statistical significance. Statistical analysis was performed using Prism software (GraphPad Software, 6 or higher version). In all instances where multiple comparisons were made, multiplicity-adjusted *P*-values were reported and were calculated with a family-wise alpha of 0.05. The results of every comparison are shown in the figures, except where noted in the figure legend. Values shown on the graphs are mean values with standard errors.

## Data Availability

The data set presented in Fig. 1D is available on the figshare.com platform: https://doi.org/10.6084/m9.figshare.29611823.v1

## SUPPLEMENTAL MATERIAL

Fig. S1A mbio.01871-25-s0001.tiff Phenotypic characterization of iPSC-derived sensory neurons.

Fig. S1B mbio.01871-25-s0002.tiff Phenotypic characterization of iPSC-derived sensory neurons.

Fig. S1C mbio.01871-25-s0003.tiff Phenotypic characterization of iPSC-derived sensory neurons.

Fig. S1 legend mbio.01871-25-s0004.docx Legend for Fig. S1.

Fig. S1D mbio.01871-25-s0005.tiff Phenotypic characterization of iPSC-derived sensory neurons.

Fig. S1E mbio.01871-25-s0006.tiff Phenotypic characterization of iPSC-derived sensory neurons.

Fig. S2 mbio.01871-25-s0007.tif Viral IE transcript ICP27 levels during latency establishment.

Fig. S1F mbio.01871-25-s0008.tiff Phenotypic characterization of iPSC-derived sensory neurons.

Fig. S2 legend mbio.01871-25-s0009.docx Legend for Fig. S2.

ASM does not own the copyrights to Supplemental Material that may be linked to, or accessed through, an article. The authors have granted ASM a non-exclusive, world-wide license to publish the Supplemental Material files. Please contact the corresponding author directly for reuse.
