# Supplementary figures and images for: Validation of human sensory neurons derived from inducible pluripotent stem cells as a model for latent infection and reactivation by herpes simplex virus 1
Source: mBio. 2025 Aug 18;16(9):e01871-25. doi: 10.1128/mbio.01871-25 (PMC12421857; doi:10.1128/mbio.01871-25)

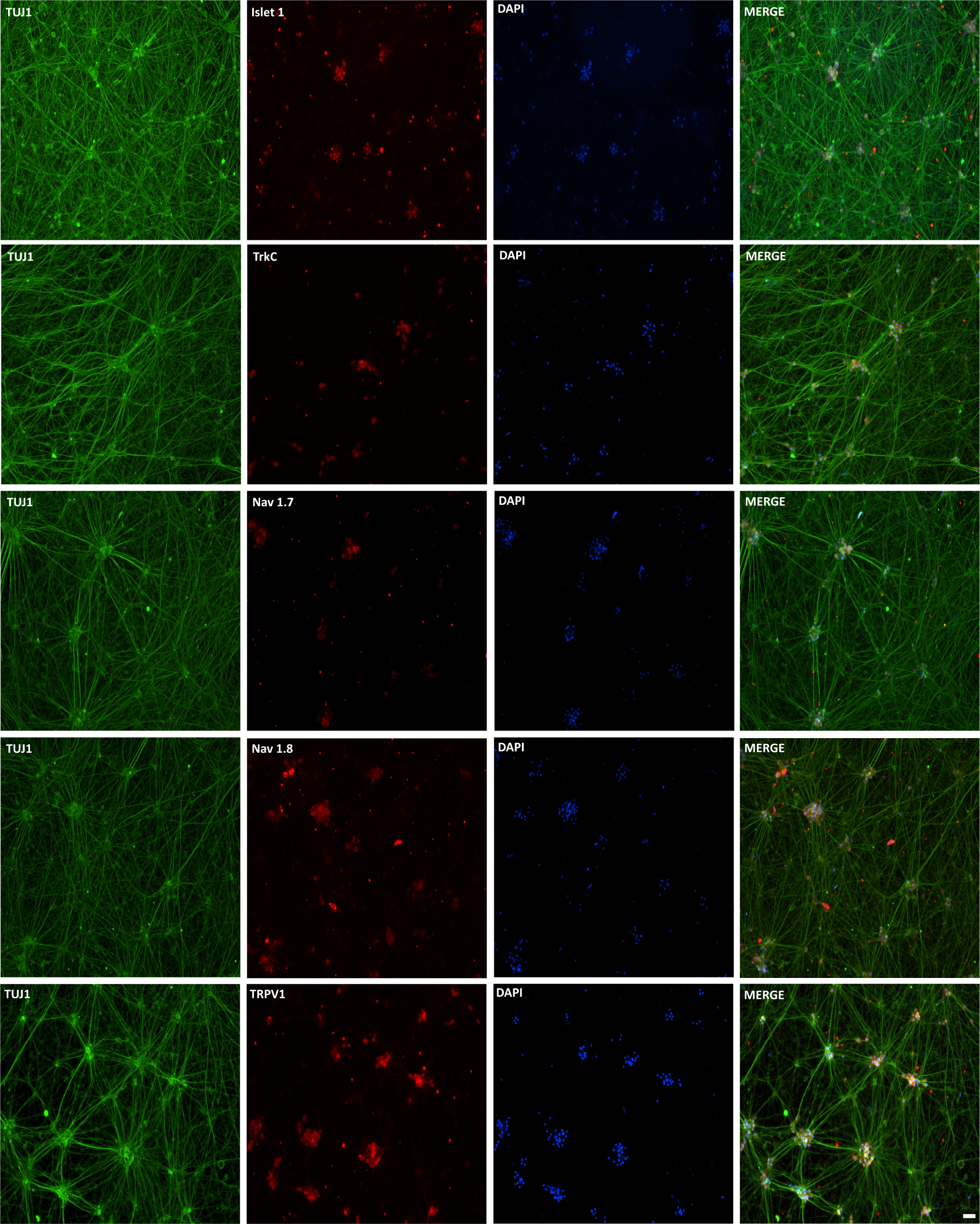

Supplement: Fig. S1A — Phenotypic characterization of iPSC-derived sensory neurons. [file mbio.01871-25-s0001.tiff]

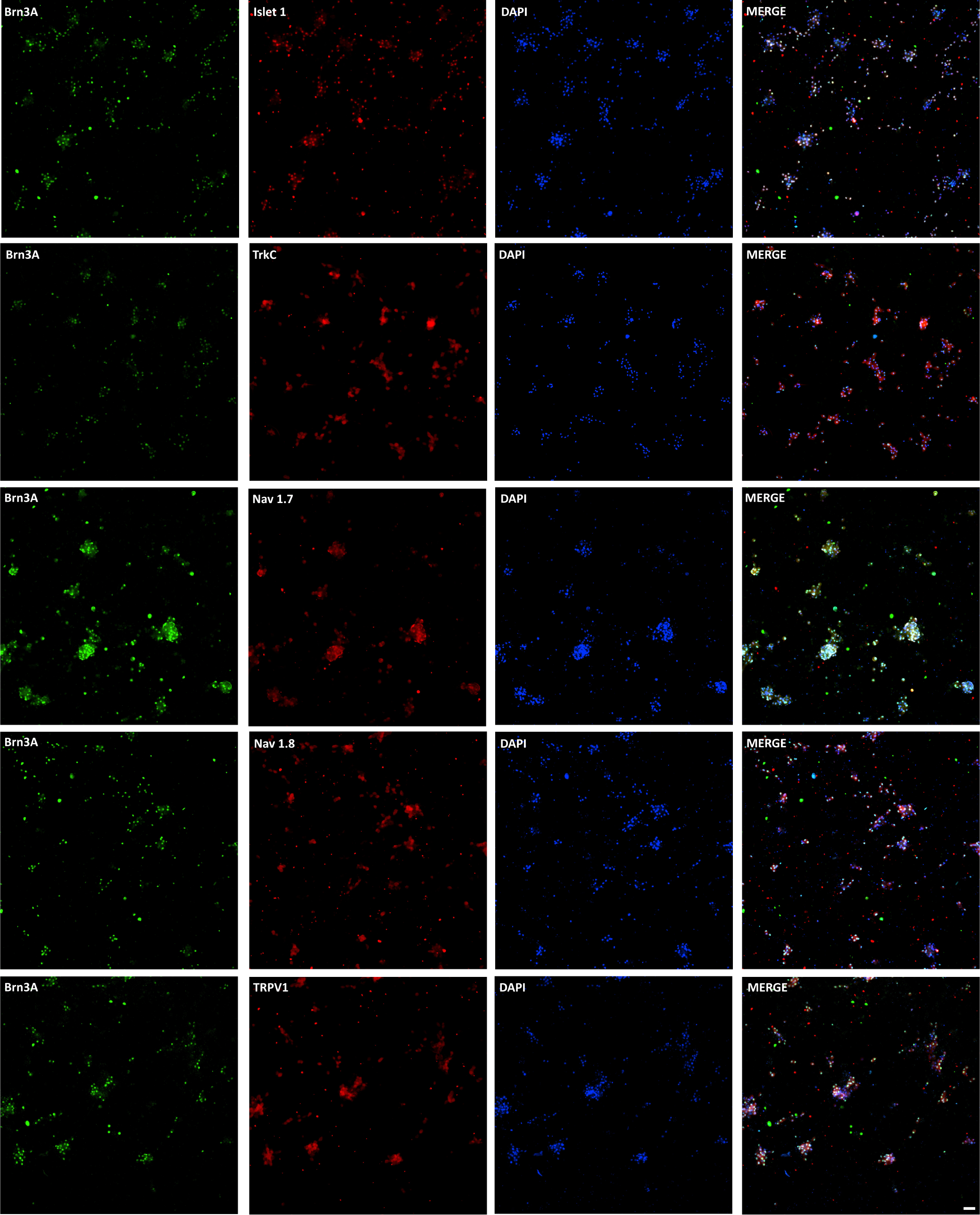

Supplement: Fig. S1B — Phenotypic characterization of iPSC-derived sensory neurons. [file mbio.01871-25-s0002.tiff]

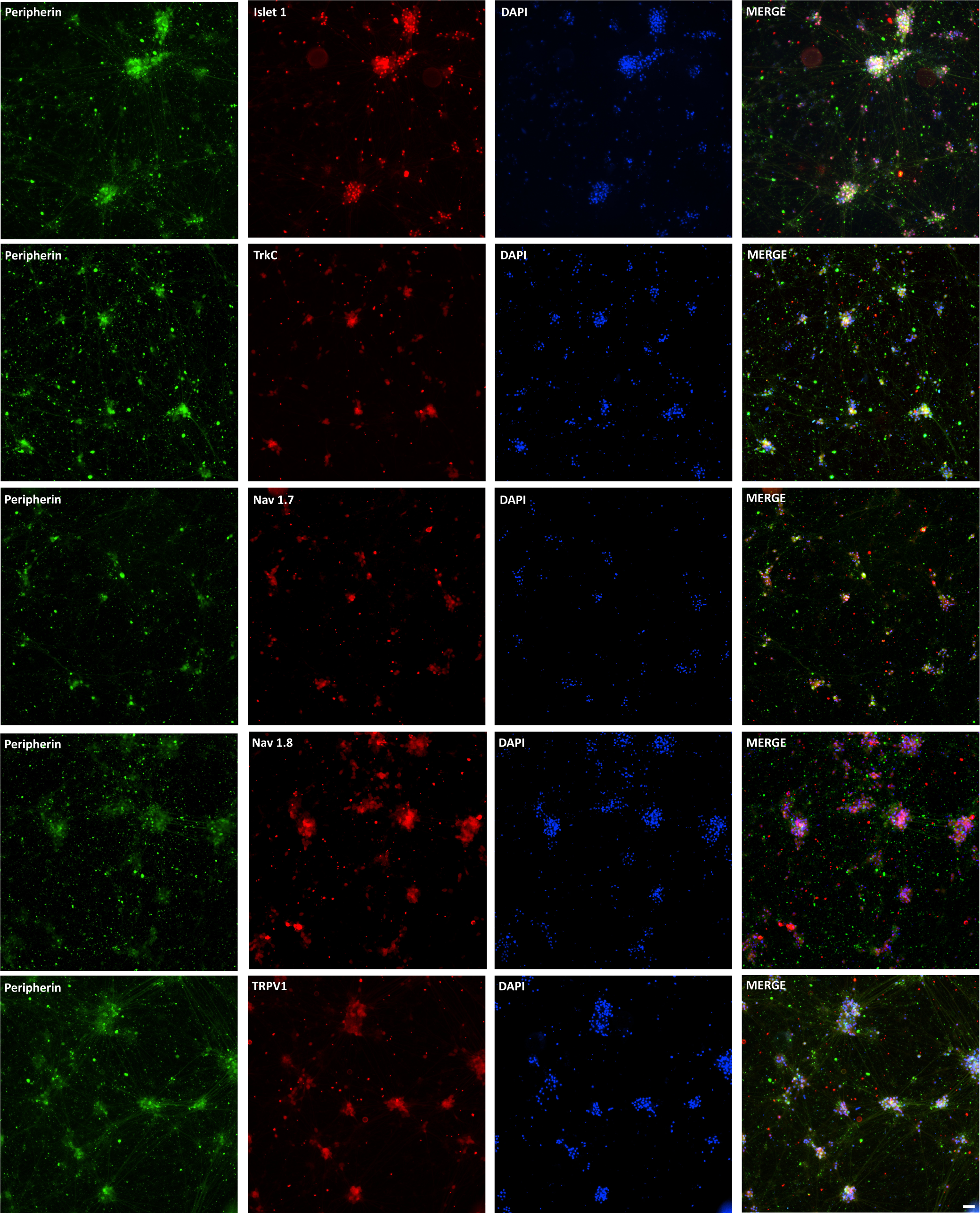

Supplement: Fig. S1C — Phenotypic characterization of iPSC-derived sensory neurons. [file mbio.01871-25-s0003.tiff]

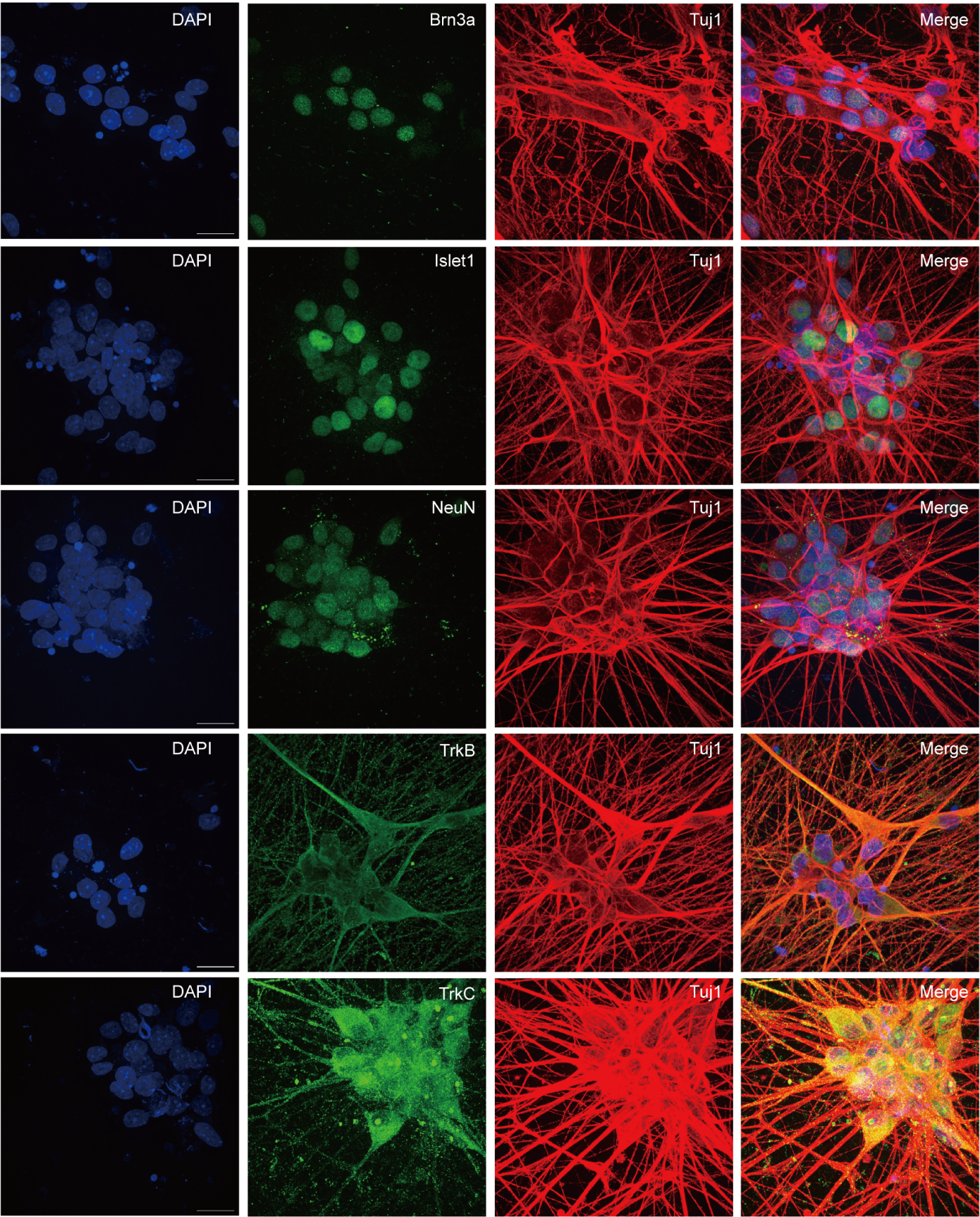

Supplement: Fig. S1D — Phenotypic characterization of iPSC-derived sensory neurons. [file mbio.01871-25-s0005.tiff]

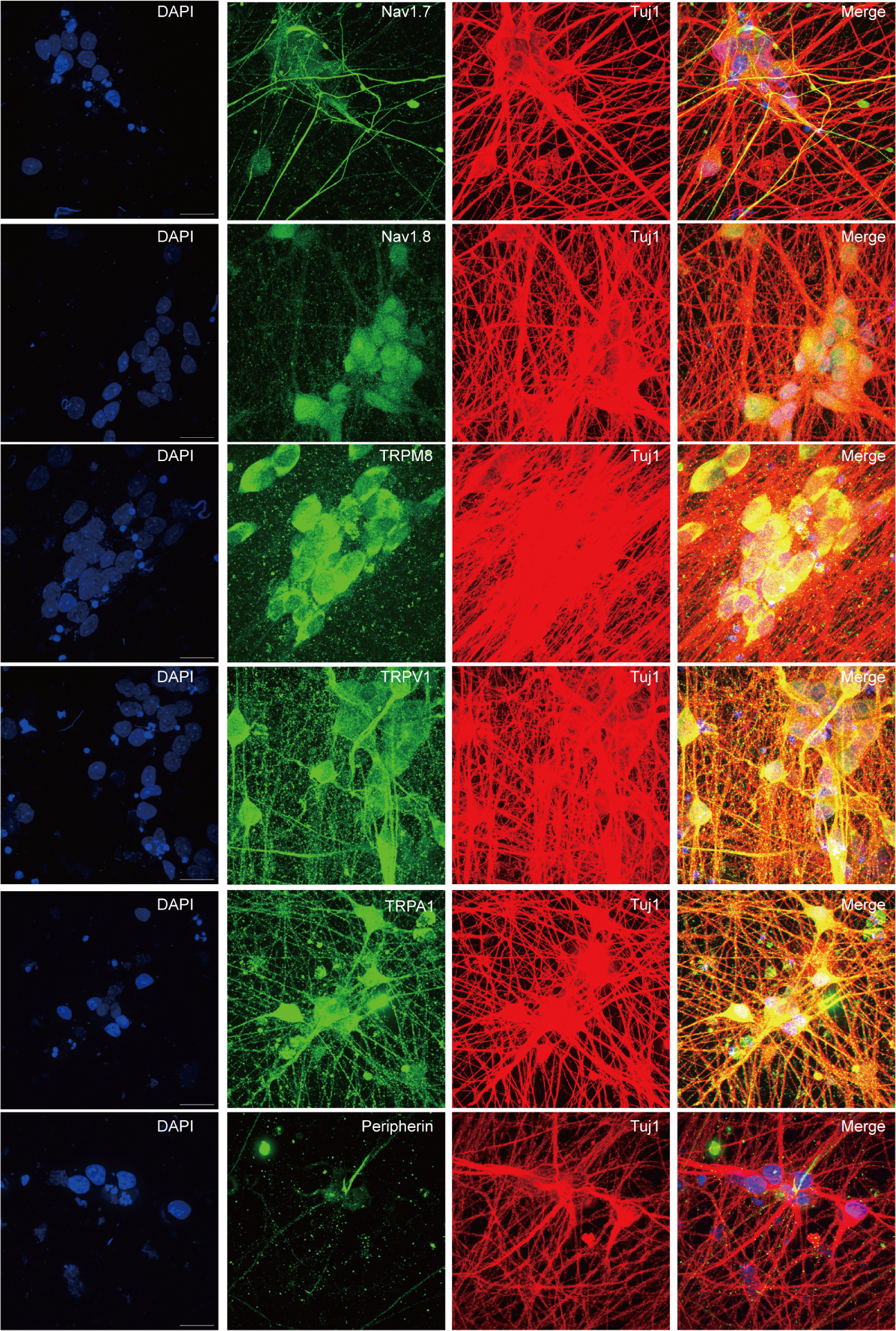

Supplement: Fig. S1E — Phenotypic characterization of iPSC-derived sensory neurons. [file mbio.01871-25-s0006.tiff]

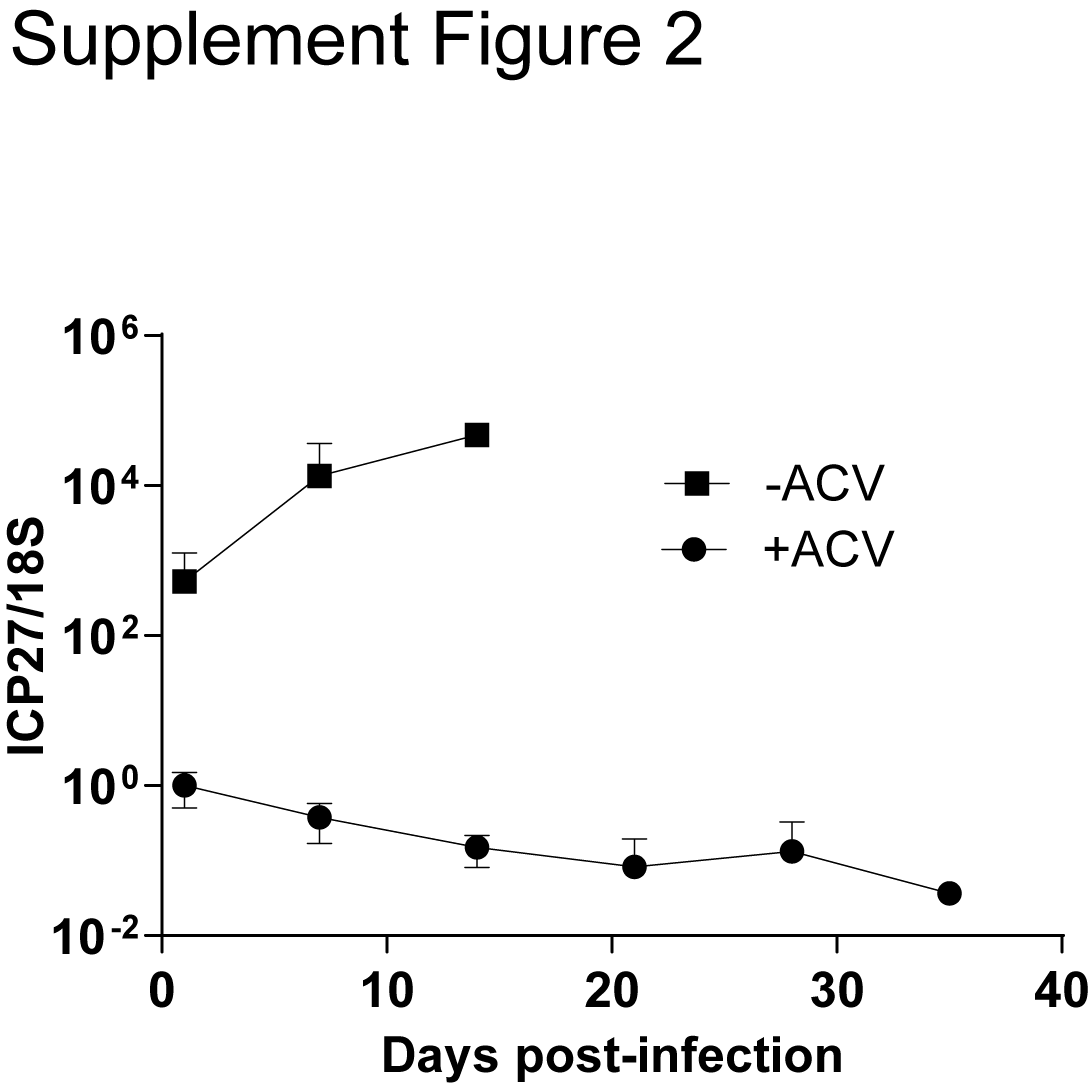

Supplement: Fig. S2 — Viral IE transcript ICP27 levels during latency establishment. [file mbio.01871-25-s0007.tif]

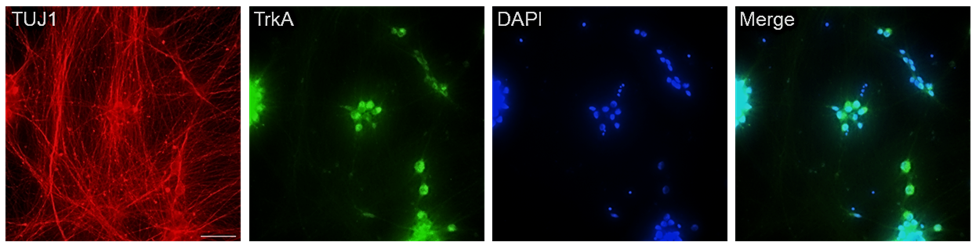

Supplement: Fig. S1F — Phenotypic characterization of iPSC-derived sensory neurons. [file mbio.01871-25-s0008.tiff]
