# Supplementary material for: Validation of human sensory neurons derived from inducible pluripotent stem cells as a model for latent infection and reactivation by herpes simplex virus 1
Source: mBio. 2025 Aug 18;16(9):e01871-25. doi: 10.1128/mbio.01871-25 (PMC12421857; doi:10.1128/mbio.01871-25)
Supplement: Fig. S1 legend — Legend for Fig. S1. [file mbio.01871-25-s0004.docx]

**Supplement Figure 1. Phenotypic Characterization of iPSC-derived sensory neurons:** iPSCs were differentiated by inducing the transcription factor (NGN3: NEUROG3) with Dox and treated with small molecule inhibitors known to differentiate iPSCs into sensory neurons. The antimitotic agent 5-Fluoro-2′-deoxyuridine was used to eliminate undifferentiated cells. (A) At 21 days post differentiation, the differentiated neurons showed extensive growth of neuron-like processes that immunostained positive for beta-III Tubulin (green). Positive co-staining of sensory neuronal maker Islet 1, TrkC, Nav 1.7, Nav1.8, and TRPV1 (each shown in red) was also observed. Nuclei were stained with DAPI (blue). (B) Neurons differentiated for 21 days-stained positive for the pan sensory factor, the POU-homeodomain factor Brn3a (green) specific for peripheral somatic neurons and co-stained for sensory neuronal markers Islet 1, TrkC, Nav 1.7, NAV1.8, and TRPV1 (each shown in red). Nuclei were stained with DAPI (blue). (C) Neurons differentiated for 21 days stained positive for the type III intermediate filaments specific for the peripheral neurons (Peripherin, green) and co-stained for sensory neuronal maker Islet 1, TrkC, Nav 1.7, NAV1.8, and TRPV1 (red). Nuclei were stained with DAPI (blue), scale Bar =100µm. (D and E) Higher magnification images were taken using spinning confocal microscope. Neurons differentiated for 21 days were stained for TUJ1 (red) and co-stained for (E) Brn3a, Islet1, NeuN, TrkB, and TrkC (green), (F) Nav1.7, Nav1.8, TRPM8, TRPV1, TRPA1, and Peripherin (green). Nuclei were stained with DAPI (blue). Scale Bar =20µm. (F) Neurons differentiated for 21 days stained for TUJ1 (red) and co-stained for TrkA (green). Nuclei were stained with DAPI (blue), scale Bar =50µm.
