# Supplementary material for: Validation of human sensory neurons derived from inducible pluripotent stem cells as a model for latent infection and reactivation by herpes simplex virus 1
Source: mBio. 2025 Aug 18;16(9):e01871-25. doi: 10.1128/mbio.01871-25 (PMC12421857; doi:10.1128/mbio.01871-25)
Supplement: Fig. S2 legend — Legend for Fig. S2. [file mbio.01871-25-s0009.docx]

**Supplement Figure 2. Viral IE transcript ICP27 levels during latency establishment.**

iNGN3-derived neurons were infected with the indicated HSV-1 strains at an MOI of 1 with ACV (200 µM) and hIgG. Latent HSV-1 infection was established for 7 d in the presence of ACV. ACV and hIgG were removed and cells were harvested at the indicated time post infection to measure ICP27 transcript by RT-qPCR. The graphs show the mean values and standard deviations of biological replicates.
